# Integration of Google Earth Engine, Sentinel-2 images, and machine learning for temporal mapping of total dissolved solids in river systems

**DOI:** 10.1038/s41598-025-12548-9

**Published:** 2025-07-29

**Authors:** Eric Ariel L. Salas, Sakthi S. Kumaran, Robert Bennett, Eric B. Partee, Jason Brownknight, Kellsie Schrack, Bryant Willis

**Affiliations:** 1 Agricultural Research Development Program (ARDP), Central State University, Wilberforce, OH 45384 USA; 2 Little Miami Conservancy, Loveland, OH 45140 USA

**Keywords:** Environmental sciences, Hydrology, Ecological modelling, Freshwater ecology

## Abstract

One of the important indicators of water quality (WQ) in inland water systems is total dissolved solids (TDS). Collecting and maintaining in situ TDS data with high spatial and temporal resolution is time and money-consuming. This study highlights an advanced approach integrating Google Earth Engine (GEE), remote sensing techniques using Sentinel-2 imagery, and machine learning algorithms to map TDS in a spatially explicit manner. We extracted relevant spectral indices and used them to train machine learning models, specifically Random Forest (RF) and Support Vector Machines (SVM), to classify TDS levels across the stretch of the Little Miami River (LMR). We analyzed TDS for August, September, October, and November, and over three years, from 2020 to 2023. Results showed RF to be more effective than SVM in mapping TDS levels, with overall accuracies and Kappa coefficients up to 0.88 and 0.85, respectively, for November 2021. Further, TDS levels remained a concern, particularly in the midstream LMR sections. Temporal rainfall variations corresponded with elevated TDS levels. Areas with higher percentages of natural cover (forests and wetlands) showed greater resilience to TDS fluctuations (r = -0.632) compared with developed or barren lands (r = 0.298). Our findings provide spatial insight into the current state of TDS as well as the success of management steps taken to manage and prevent eutrophic problems in the LMR.

## Introduction

Total dissolved solids (TDS) are among the main components of the ecological balance of rivers and other aquatic ecosystems. TDS levels measure the dissolved organic and inorganic substances in water, which include salts, minerals, metals, and organic compounds^1^. These dissolved solids are determined by passing through a 2µm filter size^2^. High levels of TDS (>500 mg/L) may have adverse effects on aquatic life, water quality (WQ), and agricultural productivity due to a reduction in the survival, growth, and reproduction of marine organisms through toxicity and disease^1,3,4^. Such high levels could bring about unpleasant taste and odor and contribute to bacterial contamination development^5^. For example, a high concentration of TDS of 900 mg/L of a contributing river had caused problems such as pipe scaling and taste and odor problems for municipal water suppliers and industrial water intakes in Ohio^6^. Furthermore, when water with high TDS levels is used for irrigation, it could affect agricultural productivity by causing soil salinization and reducing crop yields and soil fertility^7^.

Enhanced monitoring strategies are necessary to reduce the need for costly and time-consuming field visits^8^. Monitoring TDS levels and taking measures to reduce them could help protect the health of aquatic organisms, ensure safe and pleasant water quality for human use, and promote sustainable agriculture. Remote sensing (RS) has been recognized as an ideal solution for monitoring WQ in freshwater systems with successful applications and without the need for constant field visits^9^. The spectral band information from satellite images (e.g., Sentinel-2) could be used to estimate the concentration of different dissolved ions in the water, which could then be used to estimate the TDS concentration. The additional spectral channels of Sentinel-2, such as the four red edge bands and the three short-wave infrared (SWIR) bands, have provided additional spectral capability to estimate WQ properties^10^. Several studies have already demonstrated the effectiveness of RS in spatiotemporal mapping of TDS levels in river systems using Sentinel-2 images^11,12^.

In the last decade, machine learning (ML) algorithms, such as Random Forest (RF) and Support Vector Machine (SVM), have been successfully used to predict and map TDS concentrations in river systems globally. A TDS mapping study of waterbodies in Brazil using Sentinel-2 images reported high accuracy for both algorithms, with RF performing better than SVM^13^. Another study in the Owabi Reservoir in Ghana found both algorithms to be effective in predicting TDS^11^; and similarly, a TDS study in the Nile Delta region of Egypt using Sentinel 2 A and 2B data resulted in an overall accuracy of 92.5%^14^. Although many of these ML models exist, they did not combine different types of variables (e.g., time- and site-specific WQ indices and properties and vegetation indices) to model TDS. Having a high-dimensional dataset is important for a complete representation of environmental data that affect the study area, especially temperature that is specific to the time and site^15^. Further, introducing a variable unique to a location ensures that ML models learn site-specific patterns and improve mapping accuracy^16^.

Many published earth science studies have used the open source Google Earth Engine (GEE) for its ability to analyze and process huge amounts of data^3,17^. The free cloud-based computing platform provides an extensive global and temporal data catalog that includes datasets such as Sentinel-2 images. It also has a browser-based code editor interface that is capable of running complex ML analysis using the GEE datasets and providing rapid results^18^. In recent years, the GEE platform has been popular for the prediction and mapping of WQ parameters including TDS^4,19^. However, no previous research has been done that has mapped, examined, and compared temporal TDS concentrations in the state of Ohio using GEE.

In this study, we proposed to expand the use of Sentinel-2 images and Google Earth Engine (GEE) to extract and map monthly (August, September, October, and November) TDS concentrations in the Little Miami River (LMR) in Southwestern Ohio from 2020 to 2023 using RF and SVM. The LMR, specifically its contributory East Fork Little Miami River, has been identified in the recent report by the Ohio Environmental Protection Agency (OEPA) as a priority project^20^. We selected the LMR because the results of empirical models indicated that the distortions and anomalies of fish, the decrease in population sizes, and the structure of different species (e.g. insects, macroinvertebrates) may be related to the decreased water quality^21,22^. Our specific objectives include: (1) developing models for estimating TDS concentrations from Sentinel-2 images, (2) identifying important predictive variables for TDS that are essential, readily available, and easily measured, and (3) generating temporal TDS maps for LMR. Given the significant variability in land use composition and precipitation patterns in our study area, understanding spatial influences on TDS is crucial. To interpret the TDS maps, we incorporated land cover classes and precipitation data to comprehensively assess the hydrological and water quality dynamics. The results obtained in this study would have significant implications for the selection of the most appropriate ML algorithm and key predictors under SVM and RF. This research is needed to acquire a comprehensive perspective of TDS dynamics in several river locations and across diverse land cover classes and hydrological conditions.

## Results

### RF and SVM performance

Our results indicated that RF was more effective than SVM in classifying and mapping TDS levels across the studied years. Higher overall accuracy (OA) values showed that RF has a higher proportion of correctly classified instances, and higher Kappa values indicated better agreement between the observed and predicted classifications beyond chance (Table 1).

**Table 1:** Overall accuracy and Kappa statistics of RF and SVM models for classifying TDS using Sentinel-2 images from 2020 to 2023.

	2020		2021		2022		2023	
	OA	Kappa	OA	Kappa	OA	Kappa	OA	Kappa
RF								
Aug	0.800	0.774	0.778	0.738	0.758	0.724	0.755	0.715
Sep	0.724	0.694	0.754	0.713	0.700	0.670	0.650	0.613
Oct	0.720	0.701	0.833	0.804	0.796	0.765	0.788	0.765
Nov	0.752	0.721	0.878	0.845	0.818	0.796	0.683	0.658
SVM								
Aug	0.600	0.551	0.522	0.480	0.588	0.546	0.611	0.573
Sep	0.586	0.543	0.538	0.477	0.550	0.504	0.600	0.561
Oct	0.600	0.576	0.611	0.545	0.739	0.702	0.625	0.595
Nov	0.609	0.571	0.722	0.677	0.636	0.591	0.458	0.436

For October 2021 and November 2021, RF performed better than SVM in terms of classification accuracy; OA was 0.833 and Kappa 0.804 for October, while for November, OA and Kappa were 0.878 and 0.845, respectively. In addition, RF performed positively during the next years, but with some fluctuations. For example, in October 2022 and October 2023, the value of OA and Kappa remained high, accounting for 0.796 and 0.765, respectively. SVM increased in August and September of 2023, with OA and Kappa equal to 0.611 and 0.573, respectively for August, and 0.600 and 0.561 for September. Compared with RF, the performance of SVM was more irregular. The OA and Kappa values for October 2022 increased to 0.739 and 0.702, respectively, but slightly decreased in 2023 to 0.625 and 0.595. Starting with November 2023, the SVM performance significantly degraded; OA went to 0.458 and Kappa to 0.436. For training vs. validation accuracy assessment, we provided the model performance metrics for TDS estimation in Appendices A and B.

### Generated TDS maps and modeled TDS

We produced TDS maps for each month of each year using the more effective algorithm, RF. We presented the probability maps for three subsections of the LMR near the Xenia (XN) sampling location upstream, the Finley (FL) location midstream, and the Milford (MF) location downstream, using defined intervals based on the distribution of the entire TDS measurements. The rest of the maps for the other intermediate sampling sites – Indian Ripple (IR), River Edge (RE), Peters (PT), Loveland (LL), and Kelleys (KL) – are in Appendices C to G.

#### TDS levels for Xenia Site (Upstream)

**Fig. 1:**
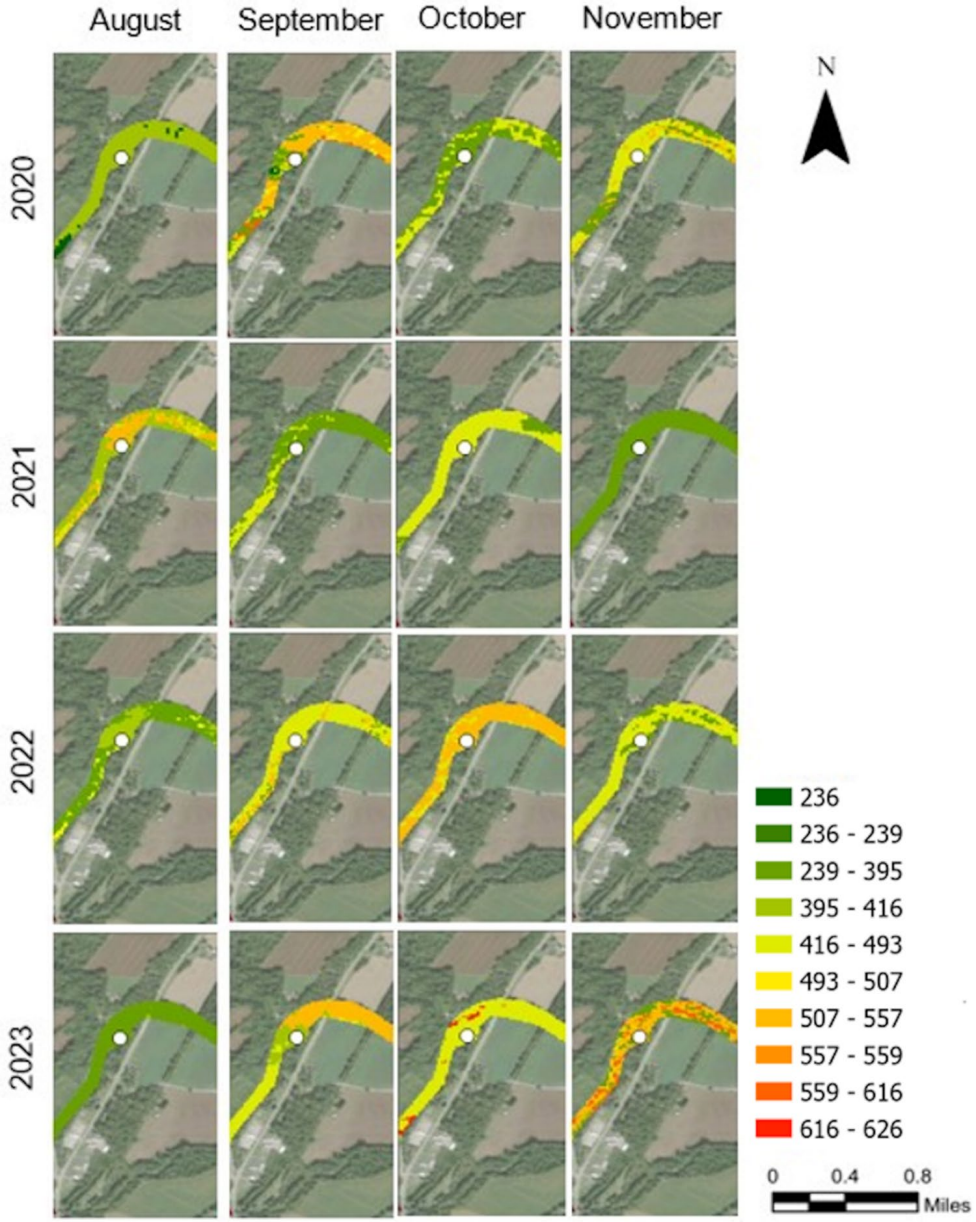
TDS (mg/L) for RF results in forms of probability maps for the upstream sampling location (XN) along the LMR. The defined TDS intervals were based on the distribution of the entire dataset of in-situ TDS measurements. The TDS map was processed in ArcGIS Pro v.3.4.0 software by Esri (source: https://www.esri.com/). The base image was provided by ESRI World Imagery (source: ESRI, Maxar, Earthstar Geographics, and the GIS USER Community).

Comparing the monthly results in Figure 1 for the upstream XN site, the results for August 2020 recorded moderate TDS levels of 236 and 416 mg/L, a trend which continued at low TDS levels in August 2022 and August 2023. The TDS in September 2020 and 2023 showed moderate levels, with values frequently exceeding 500 mg/L. September 2021 had lower TDS values, while September 2022 had moderate values, with some high concentrations in certain areas. In October, the levels were moderate during 2021 and increased in 2022 and 2023. During November, the TDS levels were moderate, except that during 2023, high levels were detected (from 559 to 626 mg/L). The yearly comparison for the XN site showed that during the year 2020, the concentration of TDS was variable with remarkable peaks, especially during September. Thereafter, it showed a trend for much more stable and low values, especially during August. Despite that, October and November 2022 and 2023 showed a trend of high TDS levels.

#### TDS levels for Finley Site (Midstream)

**Fig. 2:**
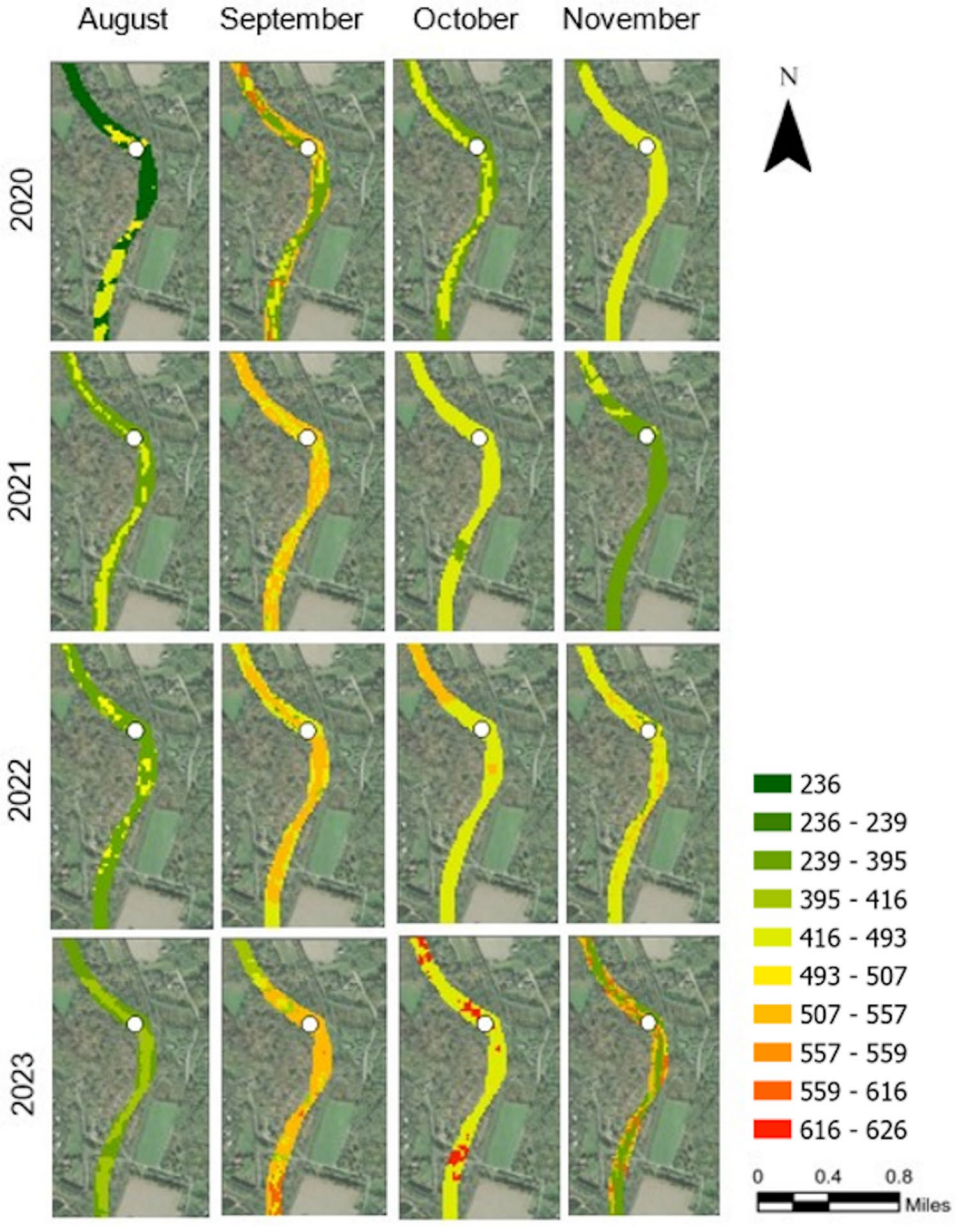
TDS (mg/L) for RF results in forms of probability maps for one of the sampling locations (FL) along the LMR. The defined TDS intervals were based on the distribution of the entire dataset of in-situ TDS measurements. The TDS map was processed in ArcGIS Pro v.3.4.0 software by Esri (source: https://www.esri.com/). The base image was provided by ESRI World Imagery (source: ESRI, Maxar, Earthstar Geographics, and the GIS USER Community).

The probability maps for TDS levels in the FL midstream section (Figure 2) showed notable variations in different years and months. In 2020, TDS levels in August were low to moderate, with values primarily in the 236 to 395 mg/L range. September exhibited increased TDS levels with more widespread areas in the 395 to 493 mg/L range. TDS was reduced in October and November. In 2021, August showed moderate levels of TDS, with a mix of values in the 395 to 493 mg/L range and some areas in the 236 to 395 mg/L range. September had higher TDS levels, with significant areas in the 493 to 557 mg/L range. October saw a mix of moderate to high TDS levels, reaching up to 493 mg/L. However, November showed a notable reduction in TDS levels that indicated lower concentrations. September, October, and November 2022 maintained high TDS levels, with a predominance of values in the 395 to 493 mg/L range and with some areas over 550 mg/L. In 2023, August started with moderate levels of TDS, while September and October saw increased levels of TDS and some regions reached up to 616 mg/L. However, November showed a mixed pattern, with some areas of high TDS levels (557 mg/L) and others showing moderate values. Overall, we observed the highest concentrations at the FL site in September 2020 and October 2023, reaching up to 616 mg/L. Generally, November had a lower trend for TDS compared to the previous months in each year, except for 2023, for which the results were mixed.

#### TDS levels for Milford Site (Downstream)

**Fig. 3:**
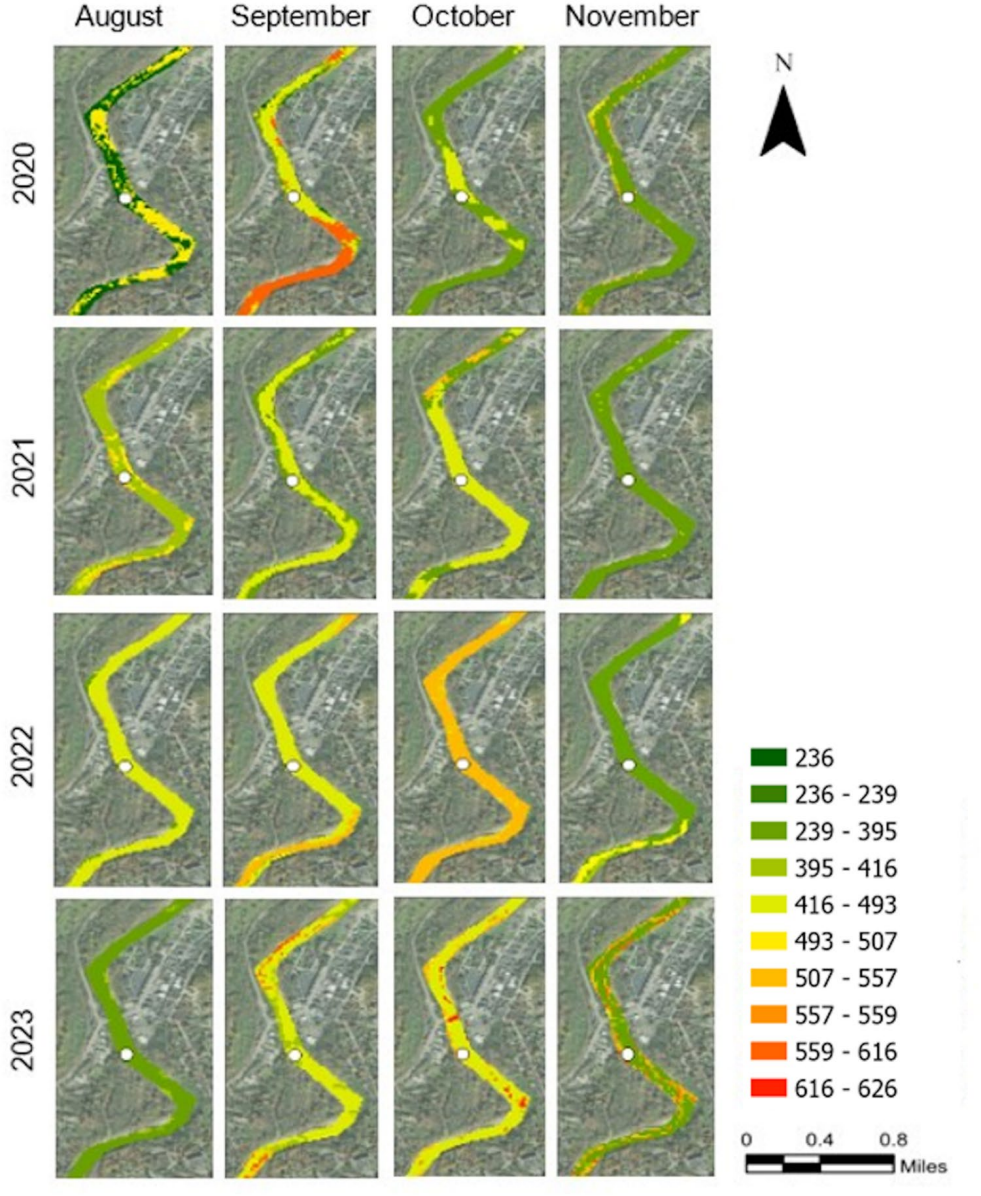
TDS (mg/L) for RF results in forms of probability maps for one of the sampling locations (MF) along the LMR. The defined TDS intervals were based on the distribution of the entire dataset of in-situ TDS measurements. The TDS map was processed in ArcGIS Pro v.3.4.0 software by Esri (source: https://www.esri.com/). The base image was provided by ESRI World Imagery (source: ESRI, Maxar, Earthstar Geographics, and the GIS USER Community).

The maps for the downstream MF site (Figure 3), revealed distinct temporal and spatial patterns in TDS levels. In 2020, TDS levels were relatively low in August (236 to 395 mg/L), with slight increases in September, while October and November showed a mixture of moderate levels (395 to 493 mg/L). In 2021, August TDS levels were higher compared to 2020, and some areas even reached 507 mg/L. September and October showed similar levels, but by November, we observed a notable reduction in TDS values (236 to 395 mg/L). In August 2022, TDS levels increased and this trend continued through September and October. November again showed a reduction (236 to 395 mg/L), similar to previous years. For 2023, high TDS levels were observed, particularly in September and October, where some areas reached 616 mg/L. However, November once again showed a decrease in TDS values (236 to 395 mg/L range).

#### TDS levels for other intermediate sites

The TDS probability maps from the other five intermediate sampling locations revealed distinct spatial and temporal patterns during the 2020-2023 monitoring period (Appendices B to F). Each location – IR, RE, PT, LL, and KL – demonstrated unique concentration profiles, ranging from 236 mg/L to over 616 mg/L. IR showed an increase in TDS over time, starting with moderate levels (236 to 416 mg/L) in 2020 and increasing to concerning levels (507 to 559 mg/L) at the end of 2023. RE exhibited considerable variability, with notable spikes in September 2020 and elevated concentration throughout 2023. The PT station exhibited early variability in 2020 but showed reverse seasonal patterns in 2023, beginning low in August and increasing throughout the fall. Station LL showed to be the most stable location with consistently moderate TDS levels (395 to 493 mg/L) in 2022 and 2023. The location in KL had the most extreme variations, starting in 2023 with concerning levels in August and then exceeding 616 mg/L by November.

#### Comparison of TDS levels for the LMR stretch

**Fig. 4:**
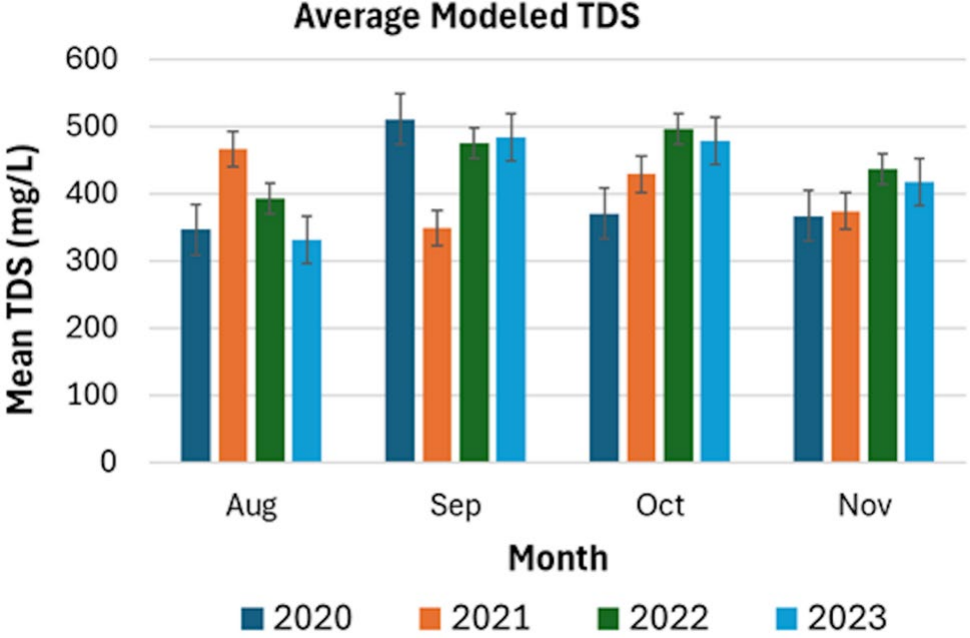
Average modeled total dissolved solids (TDS) from August to November for the years 2020 to 2023.

The average modeled TDS levels for the whole LMR stretch for each month across the years are shown in Figure 4. TDS levels in August showed a significant spike in 2021 compared to other years, which remain relatively close to each other. TDS in September is highest in 2020, 2022, and 2023, but recorded low levels in 2021. October showed high TDS values for 2022 and 2023, with a slight decrease in 2021 and a more significant drop in 2020. However, November showed consistently low TDS levels among the months, with increases observed in 2022 and 2023, but remained low for 2020. In terms of monthly variations, TDS values were observed to be higher in September and October compared to August and November. We noticed a notable spike in TDS levels in September 2020, which is significantly higher than in other years.

**Fig. 5:**
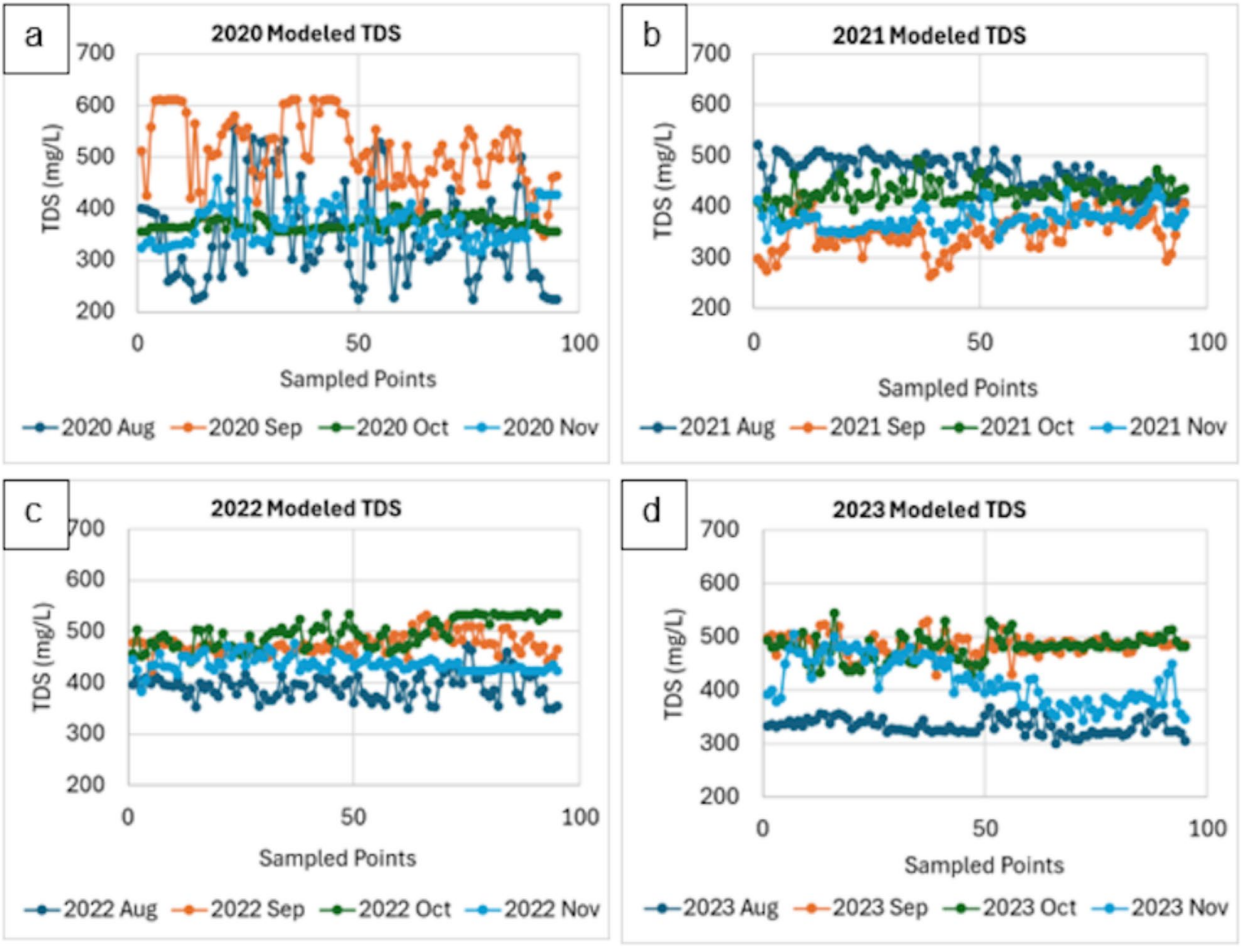
Monthly plots of the modeled total dissolved solids (TDS) for (**a**) 2020, (**b**) 2021, (**c**) 2022, and (**d**) 2023.

Figure 5 showed the monthly modeled TDS measurements from 2020 to 2023. Each plot (a-d) highlighted the variability and trends in the TDS levels across sampled points of the LMR for the respective year. We found high variability with notable peaks for 2020, especially for September with TDS values frequently exceeding 500 mg/L. The year 2021 showed lower and more stable values than September 2020. Overall, the year 2021 showed a trend towards more stability and lower peaks compared to 2020. The year 2022 also recorded lower and more stable TDS levels across all months. For 2023, we also found a trend of stability and lower TDS levels, particularly in August.

**Fig. 6:**
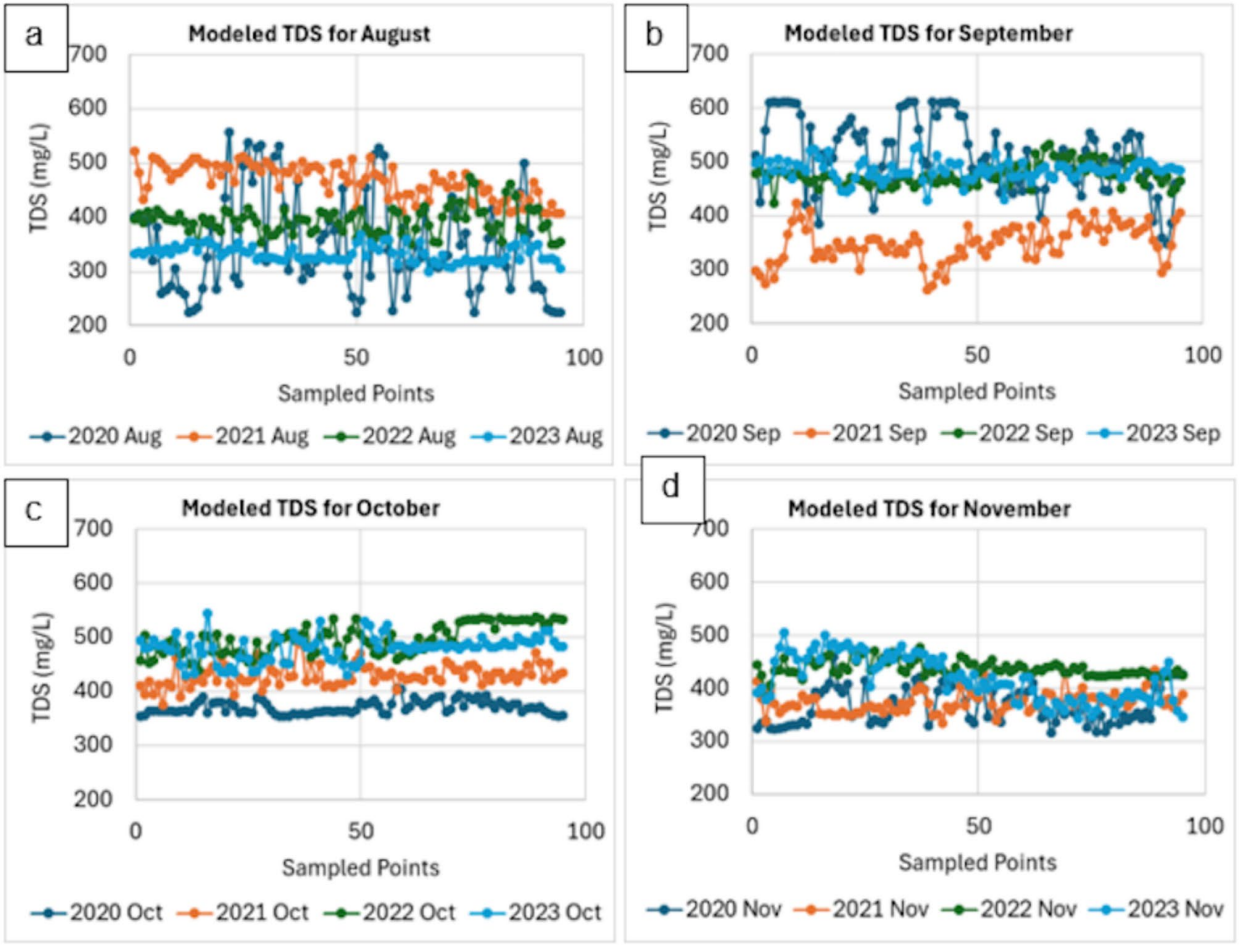
Yearly plots of the modeled total dissolved solids (TDS) for (**a**) August, (**b**) September, (**c**) October, and (**d**) November, from 2020 to 2023.

We generated yearly plots of modeled TDS in Figure 6 to compare trends for the same months in each year. For August, TDS levels fluctuated over the years. In 2020, the values fluctuated significantly from 300 to 500 mg/L. This variability was reduced in 2021, with values ranging mainly between 400 and 500 mg/L. In 2022, we observed a lower TDS level (300 to 400 mg/L), and this trend continued into 2023 with minimal fluctuations.

The results for September showed marked differences in TDS during the studied years. In 2020, TDS levels were stable; however, they often exceeded 500 mg/L. Meanwhile, TDS values varied a lot during 2021, though lower (300 to 400 mg/L). The consistency we found during 2020 was matched in 2022, wherein TDS values recorded 400 mg/L. Variations were high in 2023, with values of TDS above 600 mg/L. Unlike August and September, TDS levels for October were stable, although we observed slight increases over the years. In 2020, for example, the levels were around 400 mg/L with moderate fluctuations, while in 2021, they were higher (400 to 500 mg/L). The TDS values recorded in 2022 and 2023 were higher but steady with minimal fluctuations.

Among all months, TDS values in November were consistent and stable. November also showed more overlapping lines (Figure 6) compared to the other months, which indicated a high degree of consistency in TDS levels across the years. Overall, the TDS values for November 2020, 2021, 2022, and 2023 were relatively within a limited range between 350 and 450 mg/L. Finally, we provided tables that showed comprehensive statistical analysis, which included overall station averages (Appendix H), monthly breakdowns by station (Appendix I), and yearly trends (Appendix I).

### Important variables for mapping TDS

**Fig. 7:**
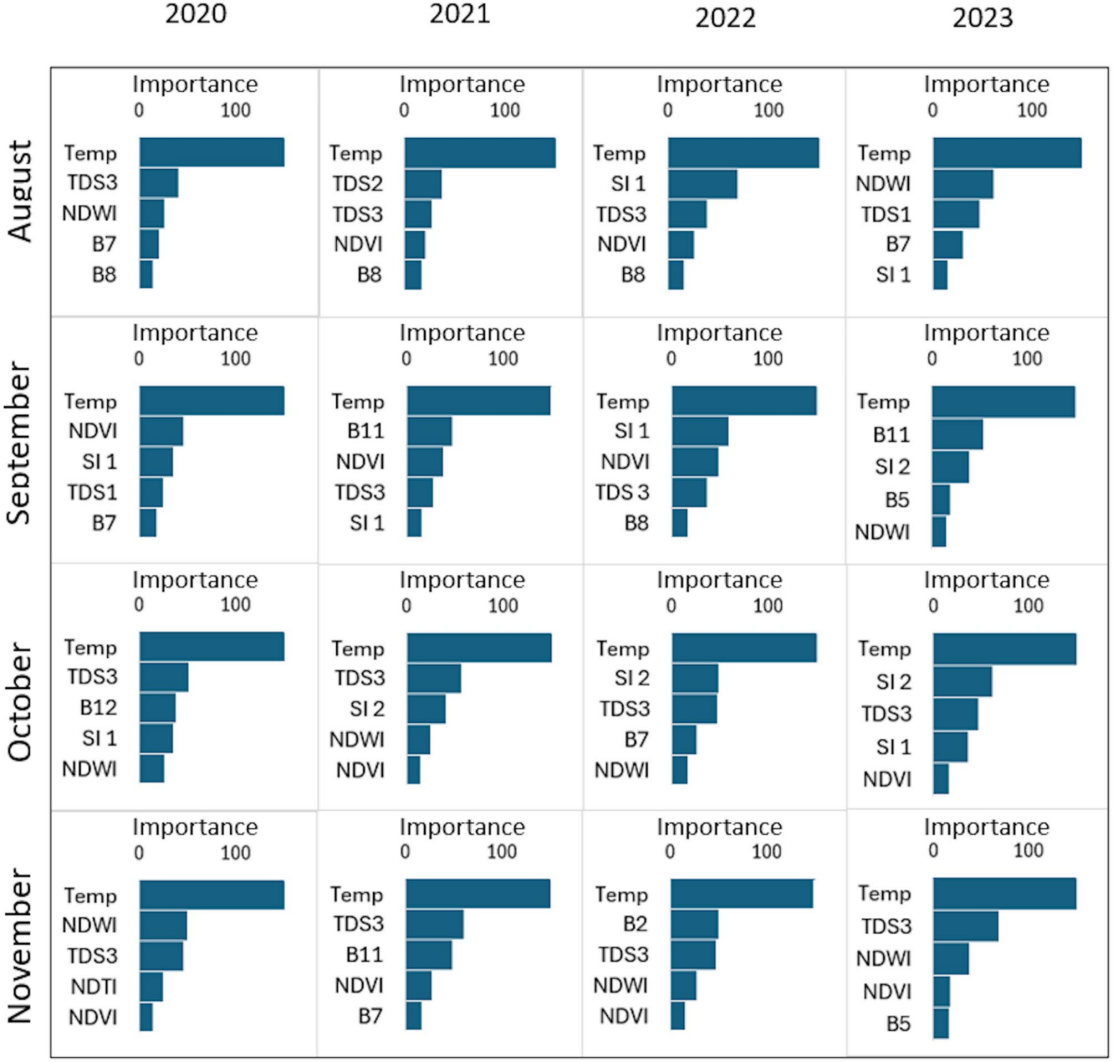
List of most important variables for classifying TDS for each studied month from 2020 to 2023.

Figure 7 shows the most important variables for classifying TDS levels across different months from 2020 to 2023. We based the importance of the variables on the Gini impurity and weight vectors^23,24^. The key variables for TDS mapping included temperature, spectral bands (e.g., B2, B5, B7, B8, B11, B12), vegetation indices (e.g., NDVI, NDWI), salinity indices, and TDS indices.

Temperature was the most consistently important variable across all months and years, highlighting its strong influence on the TDS classification. Among TDS indices, TDS3 appeared almost every month and year. For vegetation indices, NDWI and NDVI were frequently listed as important in assessing TDS levels. Salinity indices also played a role, particularly in September and October. In terms of individual spectral bands, we found that bands B2, B5, B7, B8, and B11 were important, depending on the month and year.

### Land cover and precipitation trends vs TDS

The contributing subwatersheds of the eight monitoring stations along the LMR exhibited distinct land use patterns (Table 2). The KL location had the highest proportion of woody wetlands (49.50%), while IR exhibited the highest percentage of barren land (62.59%). Sites FL and PT were dominated by forested areas, with mixed forest covering 41.84% and 28.53% of their respective areas. Agricultural land use (cultivated crops and hay/pasture) was most prominent at XN and PT, comprising 21.42% and 67.66%, respectively. Developed land, including low- and high-intensity urban areas, was particularly prevalent at IR, PT, and MF, with high-intensity development reaching up to 27.71% at PT.

The correlation matrix (Table 3) showed a strong negative correlation between forest cover and the average TDS (−0.63), while developed area has a moderately positive correlation with TDS (0.30). We found that among landuse classes, forests and developed areas were negatively correlated (−0.40), as well as forests and agriculture (−0.46). We also observed a strong negative correlation between developed and agriculture (−0.63). For wetland areas, it showed relatively weak correlations with other variables (−0.15 to 0.17).

Precipitation data (Table 4) revealed substantial interannual variability, with notable rainfall events occurring in August 2021, particularly at PT (26.47 mm) and LL (22.96 mm). In contrast, several months, including October and November of 2020 and 2021, recorded no precipitation at any station. The November 2022 event was the most widespread, with all stations receiving measurable precipitation, peaking at MF (12.32 mm). The 2023 dataset followed a similar dry trend, with only sporadic rainfall observed in November.

**Table 2:** Percent land use/land cover distribution for each monitoring station’s contributing watershed area along the Little Miami River (LMR): Xenia (XN), Indian Ripple (IR), River Edge (RE), Finley (FL), Peters (PT), Loveland (LL), Kelleys (KL), and Milford (MF).

Landuse Class	XN	IR	RE	FL	PT	LL	KL	MF
Woody Wetlands	0.94	22.27	3.36	7.81	14.99	0.24	49.50	0.89
Shrub/Scrub	0.47	5.14	4.40	9.26	44.71	3.65	29.09	3.27
Open Water	2.08	9.71	11.06	13.03	30.57	6.36	15.96	11.23
Mixed Forest	3.28	0.71	4.28	11.34	41.84	4.58	28.53	5.44
Herbaceuous	14.45	2.30	4.24	13.95	34.66	3.24	21.70	5.46
Hay/Pasture	4.54	8.58	9.51	16.87	41.37	0.58	17.46	1.09
Evergreen Forest	11.12	4.53	11.33	26.26	31.84	2.70	8.26	3.97
Emergent Herb. Wetlands	8.50	33.67	19.73	15.56	16.10	1.42	3.71	1.30
Developed, Open Space	3.68	22.30	11.56	6.62	23.26	7.50	19.83	5.24
Developed, Medium Intensity	1.93	21.21	12.68	2.45	28.18	9.22	18.72	5.62
Developed, Low Intensity	2.69	23.42	14.44	3.43	25.60	7.46	19.18	3.78
Developed, High Intensity	1.23	21.77	9.02	1.17	27.71	13.60	18.34	7.16
Deciduous Forest	7.47	6.69	10.82	17.71	31.39	3.41	17.50	5.02
Cultivated Crops	16.88	13.03	13.47	17.14	26.29	0.02	13.07	0.09
Barren Land	0.52	62.59	4.35	4.25	21.06	0.33	3.89	3.01

**Table 3:** Correlation coefficients between selected landuse classes and mean TDS levels.

Class	Forest	Developed	Wetland	Agriculture	Ave. TDS
Forest	1.00	−0.40	−0.15	−0.46	−0.63
Developed	−0.40	1.00	0.17	−0.63	0.30
Wetland	−0.15	0.17	1.00	−0.06	0.11
Agriculture	−0.46	−0.63	−0.06	1.00	0.24
TDS Average	−0.63	0.30	0.11	0.24	1.00

**Table 4:** Recorded precipitation (mm) amounts at eight monitoring stations – Xenia (XN), Indian Ripple (IR), River Edge (RE), Finley (FL), Peters (PT), Loveland (LL), Kelleys (KL), Milford (MF) – along the Little Miami River (LMR) from 2020 to 2023 that coincide with the dates of the Sentinel-2 images.

Year	Month	XN	IR	RE	FL	PT	LL	KL	MF
2020	Aug	0.769	1.07	2.17	0.303	0	0.114	0	0
	Sep	0.591	0.237	0.68	0.89	0.107	0.097	0.386	0.479
	Oct	0.306	0	0	0	0	0	0	0
	Nov	0	0	0	0	0	0	0	0
2021	Aug	10.595	16.97	5.183	13.439	26.467	22.957	6.869	4.447
	Sep	0	0	0	0	0	0	0	0
	Oct	0	0	0	0	0	0	0	0
	Nov	0	0	0	0	0	0	0	0
2022	Aug	0	0	0	0	0	0	0	0
	Sep	0	0	0	0	0	0	0	0
	Oct	0	0	0	0	0	0	0	0
	Nov	2.054	2.077	3.601	6.432	4.649	9.838	9.89	12.32
2023	Aug	0	0	0	0	0	0	0	0
	Sep	0	0	0	0	0	0	0	0
	Oct	0	0	0	0	0	0	0	0
	Nov	0.719	0.127	0.16	2.146	1.1	0	0.356	1.365

Forest cover was associated with a decrease in river TDS, while developed land and agriculture were associated with TDS increase (Table 5). The addition of precipitation data showed an improvement in model performance (r² = 0.65, p = 0.41). In the second model, forest cover remained associated with a decrease in TDS, while developed land showed a slight decrease in TDS, and both wetland and agriculture were associated with TDS increase.

**Table 5:** Linear regression models predicting TDS using landuse variables (model 1) and landuse plus precipitation variables (model 2).

	Estimate	Std. Error	t-value	Pr(>|t|)
Model 1: Landuse Only				
(Intercept)	442.15	21.84	20.24	0.00
Forest	−0.52	0.36	−1.44	0.22
Developed	0.04	0.31	0.13	0.91
Wetland	0.26	13.73	0.02	0.99
Agriculture	0.22	13.72	0.02	0.99
Multiple R-squared	0.40			
p-value	0.52			
Model 2: Landuse+Precipitation				
(Intercept)	470.56	27.55	17.08	0.00
Forest	−0.74	0.35	−2.09	0.13
Developed	−0.19	0.32	−0.60	0.59
Wetland	5.34	13.68	0.68	0.54
Agriculture	4.41	13.56	0.32	0.76
Multiple R-squared	0.65			
p-value	0.41			

## Discussion

We confirmed that the RF model outperformed the SVM model in classifying and mapping TDS levels in the LMR using the Sentinel-2 images from 2020 to 2023. RF achieved the best performance in November 2021. This was a significant improvement compared to other months and years, showing the robustness of RF in handling varying environmental conditions and sensor data. In contrast, SVM showed more variability in performance. The RF effectiveness of RF to its efficiency in handling complex interactions of predictor variables and accounting for non-linear relationships within the data^25–28^.

Previous studies outlined the strengths of RF in several applications to environmental and ecological data. For instance, Ham et al.^29^ and Zolfaghari et al.^30^ have indicated that RF outperformed other ML algorithms, including SVM, for the prediction of soil property values. Similarly, Colditz^31^ demonstrated the accuracy of RF for land cover classifications from satellite imagery over SVM and other algorithms. The use of multiple decision trees and the process of bootstrap aggregating further enhance the robustness and predictive power of the RF. This characteristic is particularly beneficial when dealing with Sentinel-2 images, which provide high-resolution, high-dimensionality, and multi-spectral data that are crucial for accurately assessing TDS levels^32^. In contrast, SVM, while considered one of the most powerful classifiers, is sensitive to the choice of kernel and settings of the parameters, which might explain why its performance was lower and more variable in our study^33^. The requirement of SVM for careful tuning and its sensitivity to the high dimensionality of remote sensing data could limit its applicability in dynamic environmental conditions, as evidenced by its fluctuating performance observed in our results. The consistent performance of RF in this study suggested its reliability for long-term monitoring programs. For example, the OA values of RF were notably high over multiple months in 2021, with notable peaks during October and November. This indicated that RF was relatively resilient to temporal variability in TDS levels, which is critical for developing integrated water quality monitoring and management plans. In addition, the multi-spectral data from Sentinel-2 efficiently captured the spatial heterogeneity of TDS levels with a high degree of accuracy. Our findings demonstrated the potential of integrating high-resolution satellite imagery with state-of-the-art ML techniques to enhance the activities of environmental monitoring and management along the LMR.

Among the variables included in our ML models, temperature emerged as the most important factor in predicting TDS. We expected this result because temperature influences TDS in many ways. For example, previous studies have documented the direct relationship between temperature and the solubility of minerals and salts. An increase in temperature would also increase the solubility of minerals and salts which could lead to high TDS levels^34,35^. In addition, microbial activity is mobilized when temperatures are high. In return, this would create a faster rate of organic matter decomposition and enable the release of dissolved substances^36^. Finally, temperature is also a factor in the release of ions from the weathering of rocks into surface waters^37^. All of these processes suggest that temperature not only drives the concentration levels of TDS but also controls other biogeochemical interactions within aquatic systems.

The TDS concentrations of the upper, middle, and lower reaches of LMR showed that the water quality varies in space and time. At the upstream XN site, where the landscape is dominated by cultivated crops and herbaceous land cover (Table 2), the data indicated that 2020 had the highest variation in TDS concentration, especially in September. We linked this variability to the quantity of rainfall at 0.8 mm and 0.6 mm in August and September 2020, respectively (Table 4) since TDS could tend to increase during the rainy season^38^ together with agricultural runoff from highly cropland area^39^. However, we observed a general trend toward more stable and lower TDS levels in the subsequent years, particularly in August. The case looked different in November 2022 and 2023, as TDS concentration increased. These results showed some improvement in water quality management, but the periodic spikes in TDS, especially in the latter months, remain a concern.

The stations downstream, such as LL and MF, had different surrounding landuse patterns that influenced TDS. The high-intensity developed area in LL (13.6%) could generate surface runoff due to impervious surfaces; this could cause runoff to enter the river system at concentrated levels during rainfall events, therefore increasing the level of TDS in these waterways^40^. Our correlation analysis (Table 3) supported this relationship, showing a moderate positive correlation between developed area and TDS levels (r = 0.298). On the other hand, MF, with variable precipitation patterns, had a lot of open water (11.2%) and mixed forest cover that could provide natural buffers and give stability to TDS. This contrasted with KL, with a high percentage of woody wetlands present (49.5%), which could have responded differently to rainfall events than areas with more development or agriculture. Open water and forested landscapes could moderate runoff, acting as a filter that traps sediments and dissolved solids before reaching downstream locations^41^. The buffering effect of forests was evident in Table 3. The strong negative correlation observed between forest cover and TDS levels (r = −0.632) indicated that watersheds with higher forest percentages have lower TDS concentrations. This was also the case during the rainfall event of August 2021, where TDS levels at LL and MF remained relatively stable across differing rainfall intensities of 23 mm at LL and 4.5 mm at MF. While Alnahit et al.^42^ highlighted the importance of forests in sustaining healthy streams within an agricultural watershed, our results also agreed with Hamidi^43^ and Nobre^44^ that showed land cover diversity could buffer sudden changes in water quality even when there are fluctuating rainfall patterns.

The maximum TDS values observed in 2020, 2022, and 2023 for September indicated seasonal factors concerning agricultural runoff and precipitation^45^. This trend is more pronounced in areas with intensive agricultural land use, such as XN, which has 16.7% of its area under cultivated crop cover, and areas such as IR, with 62.6% barren land cover. In October, TDS values were still high for 2022 and 2023, although they showed a decline in 2021, while in 2020 there was a marked drop. This could be related to changes in land use through reduced tillage or crop rotation schedules, along with water management that reduces runoff. The TDS spikes observed throughout September and October of all the sampling stations are likely influenced by mid-to-late summer or early autumn agricultural practices, such as an increase in fertilizer application and irrigation runoff besides urban effluents peaking in these months. Such seasonal activities often lead to an increase in the amount of TDS entering the river system^4,46^. The TDS values for November were always very low in all the sampling sites, which could be caused by reduced agricultural activities and scarcer rainfall. Except for November 2022, when noticeable precipitation occurred (ranging from 2.1 to 12.3 mm), this fact would therefore signal that rainfall events, while not at all strong during the year, could influence TDS levels at a lower magnitude than during the peak agricultural season of the year^47^.

The average modeled TDS concentrations of the whole LMR reach indicated strong interannual and intraannual spatiotemporal variability. In this respect, unexpectedly high TDS concentrations in August 2021 compared to the concentrations of the previous years are related to the extreme rainfall events that occurred on those days at various sites (ranging from 4.5 to 26.5 mm). This relationship expressed the integrated impact of different landuse types on water quality in extreme precipitation periods. These findings agreed with observations by McFeeters^48^, where similar trends were recorded for those water bodies that are prone to seasonal and anthropogenic influences.

The overall temporal analysis indicated that although TDS levels remain a concern, particularly in midstream sections of the LMR, there is some stabilization, especially in the upstream and downstream sections during August and November. Our findings suggested that the management of water quality should take into consideration not only the temporal patterns of rainfall but also the spatial distribution of land use types along the river corridor. Our data indicated that general resilience to TDS fluctuations during rainfall events was more probable within areas with a high percentage of natural land cover, for example, forests and wetlands. Whereas lands dominated by either barren soil or highly developed conditions might need additional management strategies to control the TDS loads throughout these events. Such increases in TDS over several months raised concerns that there was a need for continuation with effective management practices to alleviate these sources, which were very well-documented from previous literature as major contributors to water quality degradation in river systems^49^.

Finally, this study implied several key implications for the understanding of TDS dynamics in the LMR and the management of water quality. Better performance by the RF model suggested that this model should be preferred for all future monitoring and mapping efforts on TDS using Sentinel-2 images. Spatial and temporal insights on TDS concentrations were available from the generated TDS maps, pointing out seasons when high levels may require targeted interventions. Temporal trends in TDS concentration, especially in November 2023, may indicate the changes in land use and agricultural activities, or even in climatic conditions, which are affecting TDS levels in this river. Indeed, variables that were most relevant for the classification of TDS mapping, such as temperature, NDWI, NDVI, and several WQ indices, underlined the multifaceted nature of TDS dynamics. Spectral indices that include the red and red-edge bands such as TDS indices showed promise for WQ mapping, as also highlighted by Li^15^. These findings could be used to propose water management strategies that could reduce the impact of high TDS levels on the aquatic ecosystem and water quality.

## Methods

### Study area

**Fig. 8:**
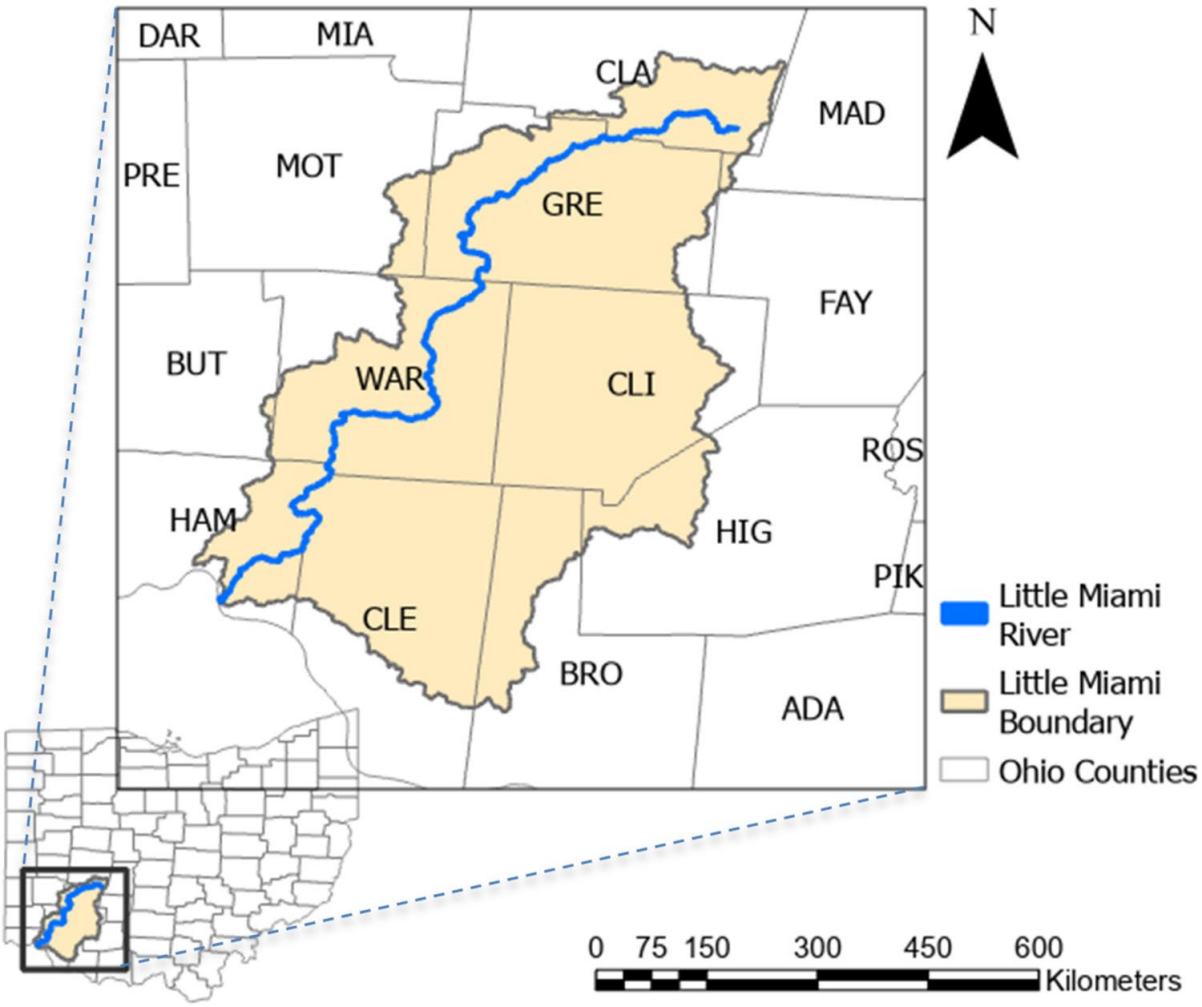
The Little Miami River (LMR) is in southwestern Ohio in the Ohio River drainage basin. This figure was created using ArcGIS Pro v.3.4.0 software by Esri (source: https://www.esri.com/).

The study area is a 111-mile (179 km) stretch of the Little Miami River in the Ohio River drainage basin in southwest Ohio (Figure 8). About 1,758 miles^2^ (4,553 km^2^) are drained by the basin in total. Eleven counties, including Greene, Warren, and Hamilton, as well as a few cities, including Xenia, Beavercreek, Wilmington, and Cincinnati, are traversed entirely or in part by the LMR. Caesar Creek, Massie Creek, and Beaver Creek are some of the larger tributaries. With a mean annual temperature that ranges from $49^{\circ }$F to $55^{\circ }$F (9.4 to 12.7 $^{\circ }$C), the study area is in a temperate continental climate. The average annual precipitation is between 35 and 43 inches (889 to 1092 mm), and it gets heavier as you move south. About one-third of that precipitation turns into surface runoff^50^. Commercial fertilizers N (nitrogen) and P (P in the form of phosphate and potash) are widely used in the basin as agriculture is the dominating land use (79%)^51^. These commercial fertilizers from row-crop farming, in addition to manure from livestock production, are the major sources of nutrients in surface and groundwater^52^.

### In-situ field monitoring sites

**Fig. 9:**
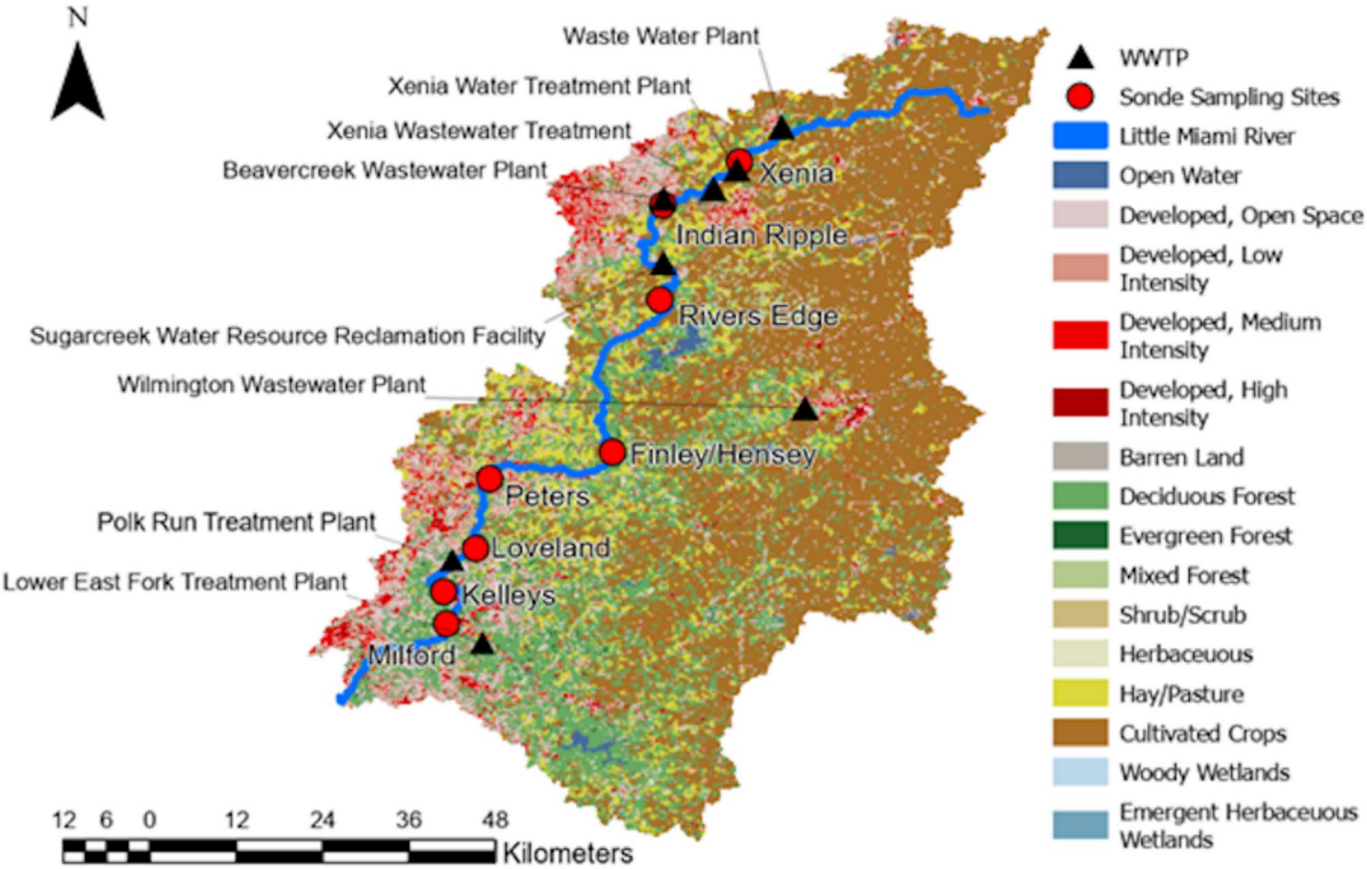
The location of the eight sampling sites along the Little Miami River overlaid on top by the land use layer for the region. The sites in the upper stretch are surrounded by cropland, while those in the lower stretch are generally located in developed areas. This figure was created using ArcGIS Pro v.3.4.0 software by Esri (source: https://www.esri.com/).

**Fig. 10:**
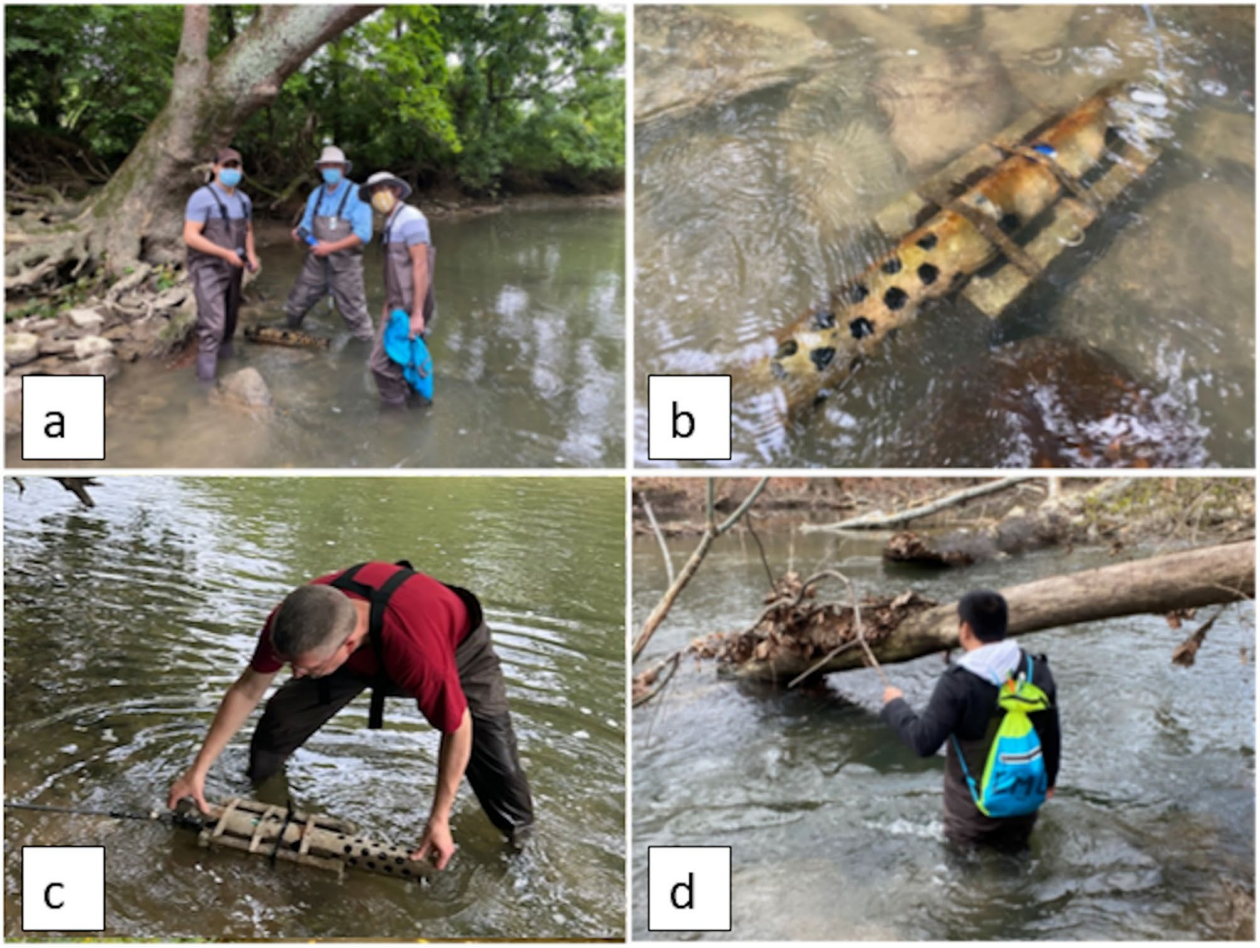
Photos showing the (**a**) authors: Salas (left), Partee (middle), and Kumaran (right) during the pre-deployment preparations of the sonde in one of the locations along the Little Miami River; (**b**) deployed sonde when submerged in the river; and regular visits to the sites by authors (**c**) Bennett and (**d**) Salas, to monitor and clean the sondes from stuck debris. Photos by the author, Salas.

At key locations along the LMR, we set up eight EXO multiparameter sondes for in-situ water quality monitoring (Figure 9), starting at the last week of July until the first week of December. We used the TDS data we collected from August to November of each year 2020, 2021, 2022, and 2023 (Table 6). July and December months had very limited data and were excluded in this study. In some cases when no data was collected from the site due to sonde malfunction, we skipped that site in the analysis and modeling. The selected sites were also near significant wastewater treatment plants (WWTP). Each sonde was protected by a metal tube and fastened to a concrete base support to prevent from being swept away by the water currents in the LMR (Figure 10). The entire assembly was also connected by a metallic cord that was fastened to a fixed structure or item (such as a tree) situated at the edge of the river. Every 15 minutes, the sondes recorded TDS data using an attached optical sensor (Table 7). The sondes also recorded the coordinates of every sampling location.

**Table 6:** Selected eight sampling sites and their locations along the Little Miami River. Water quality monitoring equipment, a sonde, is deployed in each location.

Sampling Sites	Code	River Miles (km)
Xenia/Case NP	XN	78.54 (126.4)
Indian Ripple	IR	70.87 (114.1)
River Edge	RE	58.94 (94.9)
Finley	FL	41.53 (66.8)
Peters	PT	30.47 (49.0)
Loveland	LL	23.46 (37.8)
Kelley/Livingston	KL	17.25 (27.8)
Milford/Terrace Park	MF	12.49 (20.1)

**Table 7:** Specifications of the lifetime-based EXO sensor that records TDS data.

Units	% Saturation, mg/L
Temperature (Operating/Storage)	−5 to $+50^{\circ }$C/−20 to $+80^{\circ }$C
Range	0 to 500% air sat./0 to 50 mg/L
Accuracy	0-200%: ±1% reading or 1% air sat., whichever is greater/
	200-500%: ±5% reading/0-20 mg/L: ±1% of reading or 0.1 mg/L/
	20-50 mg/L: ±5% reading
Response	T63<5 sec
Resolution	0.1% air sat./0.01 mg/L
Sensor Type	optical, luminescence lifetime

### Sampling site selection

Here are the eight selected sampling sites along the LMR.

1. Xenia (XN, Case Nature Preserve) – This Greene County site is the most upstream location where water quality reflects primarily agricultural land use in the upper watershed, and it could be compared with stage and temperature data gathered at a USGS station close to the sonde site. The data from this site, which is upstream of the main wastewater treatment discharges, could be compared to data from sites downstream.
2. Indian Ripple (IR) – This site in Greene County is downstream of the Beaver Creek Tributary, which receives two significant WWTP discharges (Xenia Waterwaste Treatment Plant and Beavercreek Wastewater Plant) and is urbanized. As one travels downstream from the XN site, this location may provide insight into the water quality effects of this tributary, the first of its kind.
3. Rivers Edge (RE) – This site borders Greene County and Warren County and is located downstream of the upper third of the watershed as well as several tributaries and subwatersheds with a mix of urban and agricultural land use. It is also downstream of all the Greene County/Yellow Springs/Xenia WWTPs.
4. Finley/Waynesville (FL Nature Preserve) – This Warren County site was chosen to potentially reflect the effects of the Caesar Creek Dam discharges as they may affect TDS and other variables, which could be used to analyze river conditions and management choices downstream in Warren County.
5. Peters Cartridge (PT) – The effects of urbanization in the Lebanon/Mason area and two WWTP discharges could be seen at this site in Warren County.
6. Loveland (LL) – Similar to the RE site, the LL site in Warren County/Hamilton County/Clermont County line could address questions that Hamilton County/Clermont County/Cincinnati staff and officials might have about the quality of the Little Miami as it enters their jurisdiction.
7. Kelley/Miamiville (KL, Camp Livingston) – Cincinnati staff may be particularly interested in the data from KL site in Hamilton/Clermont County as they consider any potential effects from the Sycamore WWTP.
8. Milford (MF, Terrace Park) - This site in Hamilton/Clermont County would presumably reflect the most downstream conditions before the mouth of the East Fork (the Little Miami’s largest subwatershed comprised of a mix of agricultural/urban landscape in addition to the East Fork dam release and several WWTP discharges).

### Sentinel-2 satellite images

We used the Sentinel-2 multispectral images available from the European Space Agency (ESA) Sentinels Scientific Data Hub (https://scihub.copernicus.eu/) to map the LMR river and allow for faster temporal analysis of TDS. Sentinel-2 has 13 spectral bands: four bands at 10 m resolution, six bands at 20 m resolution and three bands at 60 m spatial resolution. The orbital swath width is 290 km. We chose Sentinel-2 because of its optimal combination of spatial resolution, spectral resolution, and temporal resolution. By using Sentinel-2, we maximized its spatial resolution and took advantage of its spectral capabilities, such as the red-edge band. In this work, we used the associated cloud-free, monthly Sentinel-2 level 2 A scenes taken from August to November, from 2020 to 2023 that are available for our field sampling locations. We resampled all bands that were 20 m resolution to 10 m to maintain consistency with the four native bands (band 2 in blue, band 3 in green, band 4 in red, and band 8 in NIR). We also made sure that we used the field campaign dataset that corresponded to the day and time of image acquisition.

Using Sentinel-2 images, we delineated the concentrations of TDS only to the LMR by using spectral water indices such as Automated Water Extraction Index (no shadow) (AWEInsh)^53^ as well as the Sentinel Water Mask (SWM)^54^. The process of delineation is detailed in Salas et al.^55^. For parts of the river that are narrow (less than one pixel) that prevented the algorithm from extracting them, we manually intervened by adjusting the river width using high spatial resolution images from Google Earth as base maps. Although manual feature extraction is subjective, we believe that the intervention has clear and methodological benefits over automated classification methods^56^. We used Google Earth images as auxiliary datasets to enhance post-processing, fill in missing patches, and improve the accuracy of the final classification product^57^.

### RF and SVM models

We utilized RF and SVM as ML algorithms to find the non-linear relationships between various covariates and the TDS field data, and to map the TDS concentrations^28,58^ inside the GEE platform. RF and SVM are popular algorithms for mapping rivers and other surface waters, including water quality parameters using remote sensing data^11,13^. RF and SVM provide a way to select important covariates based on changes in prediction accuracy when variables are added or deleted from models.

RF is a non-parametric supervised classifier that uses a Classification and Regression Tree (CART) through bagging, where it randomly picks a set of features and creates a classifier with a bootstrapped sample of the training data to grow a tree^28^. With RF training data selection, it is possible that the same sample could be picked several times, whereas others may not be picked at all. Apart from RF being quite robust with highly collinear variables, the random selection process at each tree node causes low correlation among the trees and avoids overfitting^59^. SVM is also a non-parametric supervised classifier used for pattern recognition, classification, and regression analysis. SVM is robust in processing a small number of training samples, but efficient in producing accurate maps when applied as a classifier to satellite images^60,61^.

We determined the hyperparameter configuration for both ML models using a systematic optimization process. For example, we tested the RF classifier with a range of tree counts (5, 10, 20, 50, 100) and different variables per split (2, 4, 6, 8, 10) utilizing a grid search approach. We evaluated the performance using overall accuracy (OA) and kappa coefficient metrics. We observed that when we increased the number of trees beyond 10, the precision diminished. Similarly, we observed that the use of eight variables per split offered the best model generalization capability. For the SVM algorithm, we selected the RBF kernel based on its superior performance with our nonlinear spectral and index data. For model optimization, we tried various gamma values (0.1, 0.5, 1.0, 2.0) and cost parameters (1, 5, 10, 20, 50). Finally, we found that a gamma parameter of 0.5 and a cost parameter of 10 gave the highest validation accuracy, indicating robust model performance for the prediction of TDS across the LMR stretch.

### Covariates and datasets

To construct the RF and SVM models, we derived a set of covariates for each monthly image (August to November) for each year (2020 to 2023). The set of covariates included the following variables: Sentinel-2 bands: band 2 (b2, 490 nm), band 3 (b3, 560 nm), band 4 (b4, 665 nm), band 5 (b5, 705 nm), band 6 (b6, 740 nm), band 8 (b8, 842 nm), band 11 (b11, 1610 nm), band 12 (b12, 2190 nm); two vegetation indices: Normalized Difference Vegetation Index (NDVI), Modified Soil-Adjusted Vegetation Index (MSAVI); three water indices: Normalized Difference Water Index (NDWI), Water Ratio Index (WRI), Modified Normalized Difference Water Index (MNDWI); six water quality indices: Normalized Difference Turbidity Index (NDTI), Salinity Indices (SI-1 and SI-2), Total Dissolved Solids indices (TDS-1, TDS-2, and TDS-3). (Table 8). The spectral bands used by these specific TDS, salinity, and turbidity indices are sensitive to the optical properties of water that are affected by changes of TDS concentrations. Thus, we based the selection of our covariates from previous studies that have applied ML algorithms to remote sensing data to classify water quality parameters, including TDS^59,62–66^.

We utilized a specific range of TDS dataset that corresponded to the time of acquisition of the satellite images. When more than one satellite image is available in a month, the images are averaged to create a monthly composite. Then the field TDS data for that month would be averaged from dates corresponding to those clear image acquisitions to train the monthly model. Pairing the dates and time relatively close for the TDS data and satellite images could help ensure reliability in our analysis^67^. We manually checked the TDS values for abnormal fluctuations within the period. Before running the models, we split the final dataset into training and testing sets. We used a splitting criterion of 70–30, where 70% of the sample data was used for calibration and 30% for validation. Our independent application of the 70-30 hold-out assessment in each of the 16 monthly TDS models, and then applied over the four-year study period provided us with a degree of confidence and consistency of the method.

**Table 8:** Apart from the individual Sentinel-2A bands, here is the list of spectral indices used as variables in the RF and SVM models: b2 = blue, b3 = green, b4 = red, b5 = red-edge, b8 = NIR.

Covariates	Code	Equation	Reference
Normalized Difference Vegetation Index	NDVI	$\frac{b8-b4}{b8+b4}$	Tucker, 1979^68^
Normalized Difference Water Index	NDWI	$\frac{b3-b8}{b3+b8}$	Gao, 1996^69^
Modified Soil-Adjusted Vegetation Index	MSAVI	$\frac{2b8+1-\sqrt{(2b8+1)^2-8(b8-b4)}}{2}$	Qi, 1994^70^
Modified Normalized Difference Water Index	MNDWI	$\frac{b3-b12}{b3+b11}$	Xu, 2006^71^
Normalized Difference Turbidity Index	NDTI	$\frac{b4-b3}{b4+b3}$	Lacaux et al., 2007^62^
Salinity Index-1	SI-1	$\sqrt{b4b2}$	Maliki et al., 2020^72^
Salinity Index-2	SI-2	$\sqrt{b3^2*b8^2}$	Adjovu et al., 2022^65^
Total Dissolved Solids-1	TDS-1	$b3+b4+b5$	Tran et al., 2020^73^
Total Dissolved Solids-2	TDS-2	$b4+b5$	Mejía Ávila et al., 2022^74^
Total Dissolved Solids-3	TDS-3	$\frac{b5-b2}{b5+b2}$	Mejía Ávila et al., 2022^74^
Water Ratio Index	WRI	$\frac{b3+b4}{b8+b12}$	Mukherjee and Samuel, 2016^63^

### Google earth engine and assessment

We ran the RF and SVM models and generated TDS maps through the GEE platform (earthengine.google.com). GEE is a cloud-based platform that provides researchers with a vast collection of satellite imagery and geospatial datasets^75^. It offers a comprehensive and diverse range of data sources, including the 10-meter spatial resolution Sentinel-2. The extensive GEE data archive, coupled with its efficient data storage and processing capabilities, allowed this research seamless access to the necessary image data for analysis. By utilizing the power of GEE, we retrieved and preprocessed the Sentinel-2 imagery for all the months and years needed for this research. We calculated all spectral indices from the images using GEE. The use of GEE ensured a streamlined and efficient workflow, enabling us to leverage its vast data resources and advanced analysis capabilities for accurate TDS mapping.

We assessed the accuracy of the 16 TDS maps we produced using established metrics such as overall accuracy (OA) and kappa coefficient. These metrics provide quantitative measures of the classifier performance in correctly assessing the agreement between the predicted and reference class labels^76^. Finally, by utilizing the training and testing data splitting approach, we evaluated the accuracy of the RF and SVM classifiers and compared their mapping performance.

### TDS vs. rainfall vs. landuse

We calculated the contributory area for each sampling station within the watershed and determined the land cover classes using the National Land Cover Database (NLCD) dataset (https://www.usgs.gov/centers/eros/science/national-land-cover-database). We derived the percentages of different land cover classes within each contributory area to assess their influence on water quality. We also collected precipitation data from eight monitoring stations along the LMR for the period 2020–2023, aligning it with the dates of Sentinel-2 image acquisition. We used these datasets to analyze the relationship between TDS concentrations, rainfall events, and surrounding land use patterns. We examined spatial and temporal variations in TDS levels in the upper, middle, and lower reaches of the LMR and evaluated how different types of land use, such as cultivated crops, developed areas, and wetlands, influenced TDS fluctuations during precipitation events. We integrated statistical comparisons of trends in TDS, influences of land use, and precipitation intensities to identify the key drivers of variability in water quality and assess the effectiveness of natural and managed landscapes in moderating TDS levels.

### Limitations and future work

This study demonstrated the utility of GEE, Sentinel-2, and machine learning for TDS mapping in the LMR. However, it has several limitations that we would want to highlight in this section. We hope that in our future research we can address these limitations and expand on our current findings.

1. There is a potential for sonde malfunctions and missing data that could affect the size of the training dataset and consistency for certain dates and sampling locations, which could impact model robustness. In our case, most of the missing datasets are in July and December. These months had very limited data and were excluded from this study.
2. The models we developed are highly specific to the geographic context of the LMR. The application of our models to other river systems or other hydrological conditions would need local calibration and validation.
3. Although RF and SVM models proved effective in this study, there is always potential for further model improvement. When high-dimensional or comprehensive training datasets are involved, other deep-learning algorithms could be explored as alternative models.
4. Synthetic Aperture Radar (SAR) is sensitive to surface roughness and dielectric properties that may have an indirect correlation with TDS under specific conditions (e.g., through effects on sediment load, which can be a component of TDS). However, we did not utilize SAR in this study. For the direct retrieval of TDS, we used the more proven multispectral optical sensor, which is primarily related to dissolved constituents that affect the optical properties of water. We hope to integrate SAR for future research, as it could also mitigate cloud cover issues and provide all-weather monitoring capabilities.
5. For longer time-series analysis, Landsat imagery could have an advantage over Sentinel-2. However, the 30-m spatial resolution of Landsat could be a problem in accurately delineating rivers with narrow reaches. Another issue is the potential mixed-pixel effects caused by a coarse resolution that could affect the characterization of the TDS variability. Nonetheless, Landsat could still be effectively applied for wider rivers or for identifying broader historical trends.
6. Our approach for both RF and SVM models did not explicitly capture temporal dependencies in the data. In our future research, we would explore time series-based models such as Long Short-Term Memory (LSTM) networks to focus on the predictive modeling or the temporal dynamics of TDS. Also, we would train our model outside the GEE and implement other deep-learning models, such as XGBoost and LightGBM, to map water quality parameters.
7. We did not incorporate lagged rainfall variables (e.g., cumulative precipitation over previous days) that could affect TDS levels due to delayed runoff response. In our future analysis, we would investigate how temporal rainfall lags and hydrological connectivity could better capture upstream-downstream dynamics in TDS modeling.

## Conclusions

This study demonstrated how ML techniques could be used to map the spatial distribution of TDS concentrations in the LMR using Sentinel-2 imagery. Sentinel-2 dataset is suitable for studying the temporal changes of TDS concentrations in the LMR stretch, as shown by the overall accuracy of TDS extraction from our models. The remote sensing techniques and algorithms we used here could be applied indirectly to estimate TDS concentrations in other river systems with the right training data. However, due to the complexity of the physical, chemical, and biological processes occurring within watersheds, it is challenging to predict TDS with accuracy, particularly when conditions like nutrient conversions to rivers are unknown. Predictive models would inevitably introduce uncertainty into the research when used to map the spatio-temporal variability of TDS. Every ML technique has a unique set of benefits and drawbacks, and not one algorithm is appropriate for all applications. Given that our models are data-driven, it is crucial to use an adaptive management strategy when the data changes in the future. We recommend adjusting the decisions and running the models again after the availability of new input variables.

## Supplementary Information

Supplementary Information 1.

Supplementary Information 2.

Supplementary Information 3.

Supplementary Information 4.

Supplementary Information 5.

Supplementary Information 6.

Supplementary Information 7.

Supplementary Information 8.

Supplementary Information 9.

## Data Availability

The datasets generated during and/or analyzed during the current study are available from the corresponding author upon reasonable request.
