# Supplementary material for: Integration of Google Earth Engine, Sentinel-2 images, and machine learning for temporal mapping of total dissolved solids in river systems
Source: Sci Rep. 2025 Jul 29;15:27555. doi: 10.1038/s41598-025-12548-9 (PMC12307934; doi:10.1038/s41598-025-12548-9)
Supplement: Supplementary file 1 — Supplementary Information 1. [file 41598_2025_12548_MOESM1_ESM.docx]

|  | **2020** |  | **2021** |  | **2022** |  | **2023** |  |
| --- | --- | --- | --- | --- | --- | --- | --- | --- |
| **RF** | **R^2^** | **MAE** | **R^2^** | **MAE** | **R^2^** | **MAE** | **R^2^** | **MAE** |
| Aug | 0.98 | 0.45 | 0.99 | 0.30 | 0.99 | 0.33 | 0.99 | 0.11 |
| Sep | 0.99 | 0.46 | 0.99 | 0.12 | 0.99 | 0.19 | 0.99 | 0.15 |
| Oct | 0.99 | 0.48 | 0.99 | 0.45 | 0.99 | 0.33 | 0.99 | 0.20 |
| Nov | 0.99 | 0.43 | 0.99 | 0.29 | 0.99 | 0.44 | 0.99 | 0.46 |
| **SVM** |  |  |  |  |  |  |  |  |
| Aug | 0.98 | 0.35 | 0.99 | 0.70 | 0.99 | 0.79 | 0.99 | 0.68 |
| Sep | 0.98 | 0.68 | 0.99 | 0.68 | 0.99 | 0.57 | 0.99 | 0.46 |
| Oct | 0.99 | 0.76 | 0.99 | 0.41 | 0.99 | 0.50 | 0.99 | 0.60 |
| Nov | 0.99 | 0.44 | 0.99 | 0.47 | 0.99 | 0.88 | 0.99 | 0.75 |

**Appendix A.** Model performance metrics for TDS estimation: training vs. validation accuracy assessment.
