# Supplementary material for: Integration of Google Earth Engine, Sentinel-2 images, and machine learning for temporal mapping of total dissolved solids in river systems
Source: Sci Rep. 2025 Jul 29;15:27555. doi: 10.1038/s41598-025-12548-9 (PMC12307934; doi:10.1038/s41598-025-12548-9)
Supplement: Supplementary file 2 — Supplementary Information 2. [file 41598_2025_12548_MOESM2_ESM.docx]

**Appendix B.** Classification performance summaries for 2020, 2021, 2022, and 2023 (August-November)

**2020 – August | Overall Accuracy: 0.800 | Kappa: 0.774**

| **Class** | **Test Samples** | **Correct Predictions** | **Class Accuracy** | **Main Confusion** |
| --- | --- | --- | --- | --- |
| 1 | 2 | 2 | 1.000 | None |
| 2 | 1 | 1 | 1.000 | None |
| 3 | 2 | 2 | 1.000 | None |
| 4 | 2 | 1 | 0.500 | 1 → Class 7 |
| 5 | 2 | 1 | 0.500 | 1 → Class 3 |
| 6 | 1 | 1 | 1.000 | None |
| 7 | 2 | 2 | 1.000 | None |
| 8 | 1 | 1 | 1.000 | None |
| 9 | 2 | 1 | 0.500 | 1 → Class 10 |
| 10 | 1 | 1 | 1.000 | None |

**2020 – September | Overall Accuracy: 0.724 | Kappa: 0.694**

| **Class** | **Test Samples** | **Correct Predictions** | **Class Accuracy** | **Main Confusion** |
| --- | --- | --- | --- | --- |
| 1 | 2 | 1 | 0.500 | 1 → Class 5 |
| 2 | 1 | 1 | 1.000 | None |
| 3 | 2 | 1 | 0.500 | 1 → Class 8 |
| 4 | 2 | 2 | 1.000 | None |
| 5 | 2 | 1 | 0.500 | 1 → Class 2 |
| 6 | 1 | 1 | 1.000 | None |
| 7 | 2 | 1 | 0.500 | 1 → Class 4 |
| 8 | 1 | 0 | 0.000 | 1 → Class 3 |
| 9 | 2 | 2 | 1.000 | None |
| 10 | 1 | 1 | 1.000 | None |

**2020 – October | Overall Accuracy: 0.720 | Kappa: 0.701**

| **Class** | **Test Samples** | **Correct Predictions** | **Class Accuracy** | **Main Confusion** |
| --- | --- | --- | --- | --- |
| 1 | 2 | 1 | 0.500 | 1 → Class 6 |
| 2 | 1 | 1 | 1.000 | None |
| 3 | 2 | 2 | 1.000 | None |
| 4 | 2 | 1 | 0.500 | 1 → Class 9 |
| 5 | 2 | 1 | 0.500 | 1 → Class 7 |
| 6 | 1 | 0 | 0.000 | 1 → Class 1 |
| 7 | 2 | 2 | 1.000 | None |
| 8 | 1 | 1 | 1.000 | None |
| 9 | 2 | 2 | 1.000 | None |
| 10 | 1 | 1 | 1.000 | None |

**2020 – November |Overall Accuracy: 0.752 | Kappa: 0.721**

| **Class** | **Test Samples** | **Correct Predictions** | **Class Accuracy** | **Main Confusion** |
| --- | --- | --- | --- | --- |
| 1 | 2 | 2 | 1.000 | None |
| 2 | 1 | 1 | 1.000 | None |
| 3 | 2 | 1 | 0.500 | 1 → Class 8 |
| 4 | 2 | 2 | 1.000 | None |
| 5 | 2 | 1 | 0.500 | 1 → Class 4 |
| 6 | 1 | 1 | 1.000 | None |
| 7 | 2 | 1 | 0.500 | 1 → Class 10 |
| 8 | 1 | 1 | 1.000 | None |
| 9 | 2 | 2 | 1.000 | None |
| 10 | 1 | 1 | 1.000 | None |

**2021 – August | Overall Accuracy: 0.778 | Kappa: 0.738**

| **Class** | **Test Samples** | **Correct Predictions** | **Class Accuracy** | **Main Confusion** |
| --- | --- | --- | --- | --- |
| 1 | 2 | 2 | 1.000 | None |
| 2 | 1 | 1 | 1.000 | None |
| 3 | 2 | 1 | 0.500 | 1 → Class 6 |
| 4 | 2 | 2 | 1.000 | None |
| 5 | 2 | 1 | 0.500 | 1 → Class 9 |
| 6 | 1 | 1 | 1.000 | None |
| 7 | 2 | 2 | 1.000 | None |
| 8 | 1 | 1 | 1.000 | None |
| 9 | 2 | 1 | 0.500 | 1 → Class 2 |
| 10 | 1 | 1 | 1.000 | None |

**2021 – September | Overall Accuracy: 0.754 | Kappa: 0.713**

| **Class** | **Test Samples** | **Correct Predictions** | **Class Accuracy** | **Main Confusion** |
| --- | --- | --- | --- | --- |
| 1 | 2 | 1 | 0.500 | 1 → Class 4 |
| 2 | 1 | 1 | 1.000 | None |
| 3 | 2 | 2 | 1.000 | None |
| 4 | 2 | 1 | 0.500 | 1 → Class 7 |
| 5 | 2 | 2 | 1.000 | None |
| 6 | 1 | 1 | 1.000 | None |
| 7 | 2 | 1 | 0.500 | 1 → Class 10 |
| 8 | 1 | 1 | 1.000 | None |
| 9 | 2 | 2 | 1.000 | None |
| 10 | 1 | 0 | 0.000 | 1 → Class 5 |

**2021 – October | Overall Accuracy: 0.833 | Kappa: 0.804**

| **Class** | **Test Samples** | **Correct Predictions** | **Class Accuracy** | **Main Confusion** |
| --- | --- | --- | --- | --- |
| 1 | 2 | 2 | 1.000 | None |
| 2 | 1 | 1 | 1.000 | None |
| 3 | 2 | 2 | 1.000 | None |
| 4 | 2 | 2 | 1.000 | None |
| 5 | 2 | 1 | 0.500 | 1 → Class 8 |
| 6 | 1 | 1 | 1.000 | None |
| 7 | 2 | 2 | 1.000 | None |
| 8 | 1 | 1 | 1.000 | None |
| 9 | 2 | 1 | 0.500 | 1 → Class 3 |
| 10 | 1 | 1 | 1.000 | None |

**2021 – November | Overall Accuracy: 0.878 | Kappa: 0.845**

| **Class** | **Test Samples** | **Correct Predictions** | **Class Accuracy** | **Main Confusion** |
| --- | --- | --- | --- | --- |
| 1 | 2 | 2 | 1.000 | None |
| 2 | 1 | 1 | 1.000 | None |
| 3 | 2 | 2 | 1.000 | None |
| 4 | 2 | 2 | 1.000 | None |
| 5 | 2 | 2 | 1.000 | None |
| 6 | 1 | 1 | 1.000 | None |
| 7 | 2 | 1 | 0.500 | 1 → Class 9 |
| 8 | 1 | 1 | 1.000 | None |
| 9 | 2 | 2 | 1.000 | None |
| 10 | 1 | 0 | 0.000 | 1 → Class 4 |

**2022 – August | Overall Accuracy: 0.758 | Kappa: 0.724**

| **Class** | **Test Samples** | **Correct Predictions** | **Class Accuracy** | **Main Confusion** |
| --- | --- | --- | --- | --- |
| 1 | 3 | 2 | 0.667 | 1 → Class 5 |
| 2 | 2 | 2 | 1.000 | None |
| 3 | 3 | 2 | 0.667 | 1 → Class 8 |
| 4 | 2 | 2 | 1.000 | None |
| 5 | 2 | 1 | 0.500 | 1 → Class 3 |
| 6 | 2 | 2 | 1.000 | None |
| 7 | 2 | 2 | 1.000 | None |
| 8 | 2 | 1 | 0.500 | 1 → Class 10 |
| 9 | 2 | 2 | 1.000 | None |
| 10 | 2 | 1 | 0.500 | 1 → Class 6 |

**2022 – September | Overall Accuracy: 0.700 | Kappa: 0.670**

| **Class** | **Test Samples** | **Correct Predictions** | **Class Accuracy** | **Main Confusion** |
| --- | --- | --- | --- | --- |
| 1 | 3 | 2 | 0.667 | 1 → Class 7 |
| 2 | 2 | 1 | 0.500 | 1 → Class 4 |
| 3 | 3 | 2 | 0.667 | 1 → Class 9 |
| 4 | 2 | 1 | 0.500 | 1 → Class 2 |
| 5 | 2 | 1 | 0.500 | 1 → Class 8 |
| 6 | 2 | 2 | 1.000 | None |
| 7 | 2 | 1 | 0.500 | 1 → Class 1 |
| 8 | 2 | 2 | 1.000 | None |
| 9 | 2 | 1 | 0.500 | 1 → Class 10 |
| 10 | 2 | 2 | 1.000 | None |

**2022 – October | Overall Accuracy: 0.796 | Kappa: 0.765**

| **Class** | **Test Samples** | **Correct Predictions** | **Class Accuracy** | **Main Confusion** |
| --- | --- | --- | --- | --- |
| 1 | 3 | 3 | 1.000 | None |
| 2 | 2 | 2 | 1.000 | None |
| 3 | 3 | 2 | 0.667 | 1 → Class 5 |
| 4 | 2 | 2 | 1.000 | None |
| 5 | 2 | 2 | 1.000 | None |
| 6 | 2 | 1 | 0.500 | 1 → Class 9 |
| 7 | 2 | 2 | 1.000 | None |
| 8 | 2 | 2 | 1.000 | None |
| 9 | 2 | 1 | 0.500 | 1 → Class 7 |
| 10 | 2 | 2 | 1.000 | None |

**2022 – November | Overall Accuracy: 0.818 | Kappa: 0.796**

| **Class** | **Test Samples** | **Correct Predictions** | **Class Accuracy** | **Main Confusion** |
| --- | --- | --- | --- | --- |
| 1 | 3 | 3 | 1.000 | None |
| 2 | 2 | 2 | 1.000 | None |
| 3 | 3 | 3 | 1.000 | None |
| 4 | 2 | 1 | 0.500 | 1 → Class 8 |
| 5 | 2 | 2 | 1.000 | None |
| 6 | 2 | 2 | 1.000 | None |
| 7 | 2 | 1 | 0.500 | 1 → Class 3 |
| 8 | 2 | 2 | 1.000 | None |
| 9 | 2 | 2 | 1.000 | None |
| 10 | 2 | 1 | 0.500 | 1 → Class 5 |

**2023 – August | Overall Accuracy: 0.755 | Kappa: 0.715**

| **Class** | **Test Samples** | **Correct Predictions** | **Class Accuracy** | **Main Confusion** |
| --- | --- | --- | --- | --- |
| 1 | 2 | 2 | 1.000 | None |
| 2 | 2 | 1 | 0.500 | 1 → Class 7 |
| 3 | 2 | 2 | 1.000 | None |
| 4 | 2 | 1 | 0.500 | 1 → Class 9 |
| 5 | 2 | 2 | 1.000 | None |
| 6 | 2 | 1 | 0.500 | 1 → Class 4 |
| 7 | 2 | 2 | 1.000 | None |
| 8 | 2 | 1 | 0.500 | 1 → Class 10 |
| 9 | 2 | 2 | 1.000 | None |
| 10 | 1 | 1 | 1.000 | None |

**2023 – September | Overall Accuracy: 0.650 | Kappa: 0.613**

| **Class** | **Test Samples** | **Correct Predictions** | **Class Accuracy** | **Main Confusion** |
| --- | --- | --- | --- | --- |
| 1 | 2 | 1 | 0.500 | 1 → Class 3 |
| 2 | 2 | 1 | 0.500 | 1 → Class 8 |
| 3 | 2 | 1 | 0.500 | 1 → Class 6 |
| 4 | 2 | 1 | 0.500 | 1 → Class 2 |
| 5 | 2 | 1 | 0.500 | 1 → Class 7 |
| 6 | 2 | 2 | 1.000 | None |
| 7 | 2 | 1 | 0.500 | 1 → Class 9 |
| 8 | 2 | 2 | 1.000 | None |
| 9 | 2 | 1 | 0.500 | 1 → Class 10 |
| 10 | 1 | 1 | 1.000 | None |

**2023 – October | Overall Accuracy: 0.788 | Kappa: 0.765**

| **Class** | **Test Samples** | **Correct Predictions** | **Class Accuracy** | **Main Confusion** |
| --- | --- | --- | --- | --- |
| 1 | 2 | 2 | 1.000 | None |
| 2 | 2 | 2 | 1.000 | None |
| 3 | 2 | 1 | 0.500 | 1 → Class 5 |
| 4 | 2 | 2 | 1.000 | None |
| 5 | 2 | 1 | 0.500 | 1 → Class 8 |
| 6 | 2 | 2 | 1.000 | None |
| 7 | 2 | 2 | 1.000 | None |
| 8 | 2 | 2 | 1.000 | None |
| 9 | 2 | 1 | 0.500 | 1 → Class 7 |
| 10 | 1 | 1 | 1.000 | None |

**2023 – November | Overall Accuracy: 0.683 | Kappa: 0.658**

| **Class** | **Test Samples** | **Correct Predictions** | **Class Accuracy** | **Main Confusion** |
| --- | --- | --- | --- | --- |
| 1 | 2 | 1 | 0.500 | 1 → Class 4 |
| 2 | 2 | 1 | 0.500 | 1 → Class 9 |
| 3 | 2 | 2 | 1.000 | None |
| 4 | 2 | 1 | 0.500 | 1 → Class 6 |
| 5 | 2 | 1 | 0.500 | 1 → Class 10 |
| 6 | 2 | 2 | 1.000 | None |
| 7 | 2 | 1 | 0.500 | 1 → Class 3 |
| 8 | 2 | 2 | 1.000 | None |
| 9 | 2 | 1 | 0.500 | 1 → Class 2 |
| 10 | 1 | 1 | 1.000 | None |
