# Supplementary material for: Integration of Google Earth Engine, Sentinel-2 images, and machine learning for temporal mapping of total dissolved solids in river systems
Source: Sci Rep. 2025 Jul 29;15:27555. doi: 10.1038/s41598-025-12548-9 (PMC12307934; doi:10.1038/s41598-025-12548-9)

**Appendix E**. TDS (mg/L) for RF results in forms of probability maps for the Peters (PT) sampling location along the LMR. The defined TDS intervals were based on the distribution of the entire dataset of in-situ TDS measurements. The TDS map was processed in ArcGIS Pro v.3.4.0 software by Esri (source: https://www.esri.com/). The base image was provided by ESRI World Imagery (source: ESRI, Maxar, Earthstar Geographics, and the GIS USER Community).


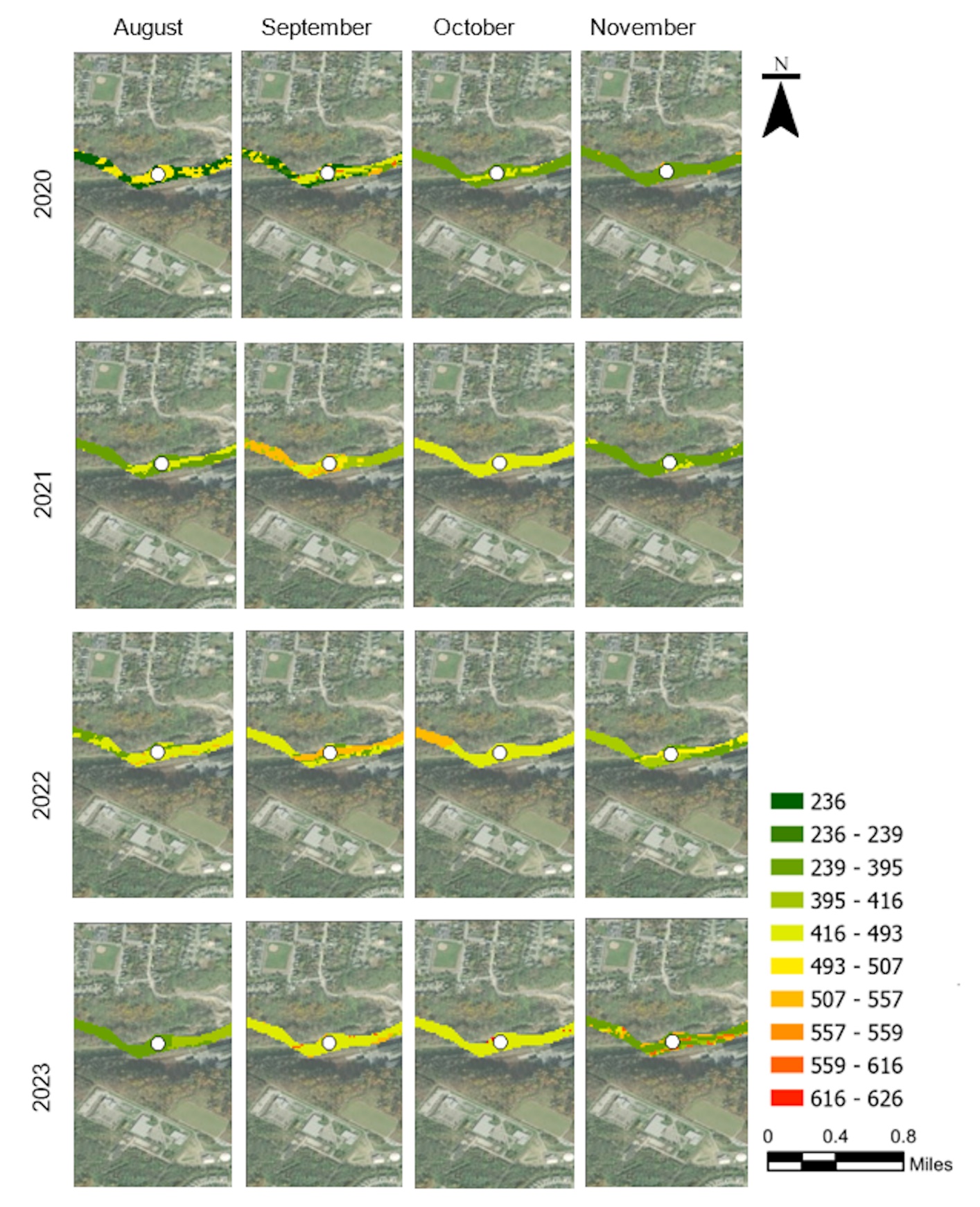

Supplement: Supplementary file 5 — Supplementary Information 5. [file 41598_2025_12548_MOESM5_ESM.docx]
