# Supplementary material for: Integration of Google Earth Engine, Sentinel-2 images, and machine learning for temporal mapping of total dissolved solids in river systems
Source: Sci Rep. 2025 Jul 29;15:27555. doi: 10.1038/s41598-025-12548-9 (PMC12307934; doi:10.1038/s41598-025-12548-9)
Supplement: Supplementary file 8 — Supplementary Information 8. [file 41598_2025_12548_MOESM8_ESM.docx]

**Appendix H**. Monthly averages of modeled TDS by station.

| **August TDS Values (mg/L)** | | |  |  |  |  |
| --- | --- | --- | --- | --- | --- | --- |
| **Station** | **2020** | **2021** | **2022** | **2023** | **Station Mean** | **Std Dev** |
| XN | 382.74 | 460.41 | 396.52 | 352.13 | 397.95 | 45.73 |
| IR | 495.39 | 512.3 | 398.6 | 336.84 | 435.78 | 85.51 |
| RE | 464.22 | 471.7 | 398.31 | 325.53 | 414.94 | 70.05 |
| FL | 291.55 | 511.02 | 371.47 | 353.55 | 381.9 | 94.17 |
| PT | 325.28 | 453.28 | 407.66 | 354.93 | 385.29 | 54.31 |
| LL | 328.73 | 454.85 | 418.59 | 308.57 | 377.68 | 67.53 |
| KL | 268.4 | 461.67 | 409.1 | 321 | 365.04 | 85.17 |
| MF | 354.59 | 426.07 | 354.75 | 318.58 | 363.5 | 43.78 |
|  |  |  |  |  |  |  |
| **September TDS Values (mg/L)** | | |  |  |  |  |
| **Station** | **2020** | **2021** | **2022** | **2023** | **Station Mean** | **Std Dev** |
| XN | 507.15 | 321.19 | 474.1 | 478.46 | 445.23 | 80.85 |
| IR | 556.81 | 338.04 | 464.34 | 503.81 | 465.75 | 89.49 |
| RE | 560.09 | 350.74 | 460.23 | 530.03 | 475.27 | 93.3 |
| FL | 469.6 | 345.44 | 488.42 | 484.53 | 446 | 70.08 |
| PT | 396.87 | 348.02 | 494.79 | 486.76 | 431.61 | 70.65 |
| LL | 482.37 | 364.46 | 505.19 | 486.33 | 459.59 | 62.53 |
| KL | 492.65 | 375.6 | 479.78 | 483.23 | 457.82 | 51.9 |
| MF | 497.29 | 380.3 | 451.58 | 470.43 | 449.9 | 48.22 |
|  |  |  |  |  |  |  |
| **October TDS Values (mg/L)** | | |  |  |  |  |
| **Station** | **2020** | **2021** | **2022** | **2023** | **Station Mean** | **Std Dev** |
| XN | 380.14 | 417.96 | 505.36 | 445.52 | 437.25 | 54.14 |
| IR | 362.48 | 418.49 | 454.72 | 459.85 | 423.89 | 44.21 |
| RE | 358.45 | 486.33 | 506.02 | 493.06 | 460.97 | 68.91 |
| FL | 385.33 | 418.44 | 469.97 | 495.86 | 442.4 | 48.73 |
| PT | 374.36 | 420.59 | 477.43 | 480.98 | 438.34 | 50.14 |
| LL | 367.77 | 448.56 | 493.53 | 484.25 | 448.53 | 54.46 |
| KL | 377.46 | 412.53 | 536.2 | 500.97 | 456.79 | 69.78 |
| MF | 375.65 | 421.52 | 530.63 | 490.93 | 454.68 | 67.86 |
|  |  |  |  |  |  |  |
| **November TDS Values (mg/L)** | | |  |  |  |  |
| **Station** | **2020** | **2021** | **2022** | **2023** | **Station Mean** | **Std Dev** |
| XN | 458.65 | 349.61 | 431.61 | 485.16 | 431.26 | 57.38 |
| IR | 415.35 | 363.1 | 470.45 | 471.52 | 430.1 | 51.95 |
| RE | 421.08 | 403.74 | 477.63 | 467.46 | 442.48 | 34.94 |
| FL | 393.94 | 369.36 | 453.74 | 421.23 | 409.57 | 34.76 |
| PT | 343.35 | 365.11 | 433.39 | 378.46 | 380.08 | 37.29 |
| LL | 380.48 | 384.71 | 429.44 | 380.47 | 393.78 | 25.49 |
| KL | 349.34 | 373.93 | 423.23 | 385.83 | 383.08 | 30.41 |
| MF | 348.26 | 376.85 | 428.93 | 383.11 | 384.29 | 33.53 |
