# Supplementary material for: Integration of Google Earth Engine, Sentinel-2 images, and machine learning for temporal mapping of total dissolved solids in river systems
Source: Sci Rep. 2025 Jul 29;15:27555. doi: 10.1038/s41598-025-12548-9 (PMC12307934; doi:10.1038/s41598-025-12548-9)
Supplement: Supplementary file 9 — Supplementary Information 9. [file 41598_2025_12548_MOESM9_ESM.docx]

**Appendix I**. Yearly averages of modeled TDS by station and the monthly averages across all stations.

**Annual TDS Averages (mg/L)**

| **Station** | **2020** | **2021** | **2022** | **2023** | **4-Year Mean** | **Std Dev** |
| --- | --- | --- | --- | --- | --- | --- |
| XN | 432.17 | 387.29 | 451.90 | 440.32 | 427.92 | 26.21 |
| IR | 457.51 | 407.98 | 447.03 | 443.00 | 438.88 | 21.32 |
| RE | 450.96 | 428.15 | 460.55 | 454.02 | 448.42 | 14.29 |
| FL | 385.11 | 411.07 | 445.90 | 438.79 | 420.22 | 26.75 |
| PT | 359.97 | 396.75 | 453.32 | 425.28 | 408.83 | 38.56 |
| LL | 389.84 | 413.15 | 461.69 | 414.90 | 419.90 | 30.12 |
| KL | 372.21 | 405.93 | 462.08 | 422.76 | 415.75 | 37.80 |
| MF | 393.95 | 401.18 | 441.47 | 415.76 | 413.09 | 21.00 |

**Monthly Averages Across All Stations**

| **Month** | **2020** | **2021** | **2022** | **2023** | **Monthly Mean** |
| --- | --- | --- | --- | --- | --- |
| August | 360.14 | 468.89 | 394.30 | 333.83 | 389.29 |
| September | 495.25 | 353.09 | 477.31 | 492.89 | 454.64 |
| October | 372.84 | 430.46 | 496.66 | 481.84 | 445.45 |
| November | 388.78 | 373.30 | 443.50 | 421.62 | 406.80 |
